# Mapping moral language on US presidential primary campaigns reveals rhetorical networks of political division and unity

**DOI:** 10.1093/pnasnexus/pgad189

**Published:** 2023-06-09

**Authors:** Kobi Hackenburg, William J Brady, Manos Tsakiris

**Affiliations:** Department of Media and Communications, The London School of Economics and Political Science, Houghton St, WC2A 2AE, London, UK; Centre for the Politics of Feelings, School of Advanced Study, University of London, Malet St, WC1E 7HU, London, UK; Department of Management and Organizations, Kellogg School of Management, Northwestern University, Clark St, 49017 Evanston, IL, USA; Centre for the Politics of Feelings, School of Advanced Study, University of London, Malet St, WC1E 7HU, London, UK; Department of Psychology, Royal Holloway, Egham Hill, TW20 0EY, London, UK

**Keywords:** moral language, political campaigns, moral foundations theory, network analysis, natural language processing

## Abstract

During political campaigns, candidates use rhetoric to advance competing visions and assessments of their country. Research reveals that the moral language used in this rhetoric can significantly influence citizens’ political attitudes and behaviors; however, the moral language actually used in the rhetoric of elites during political campaigns remains understudied. Using a data set of every tweet (N=139,412) published by 39 US presidential candidates during the 2016 and 2020 primary elections, we extracted moral language and constructed network models illustrating how candidates’ rhetoric is semantically connected. These network models yielded two key discoveries. First, we find that party affiliation clusters can be reconstructed solely based on the moral words used in candidates’ rhetoric. Within each party, popular moral values are expressed in highly similar ways, with Democrats emphasizing careful and just treatment of individuals and Republicans emphasizing in-group loyalty and respect for social hierarchies. Second, we illustrate the ways in which outsider candidates like Donald Trump can separate themselves during primaries by using moral rhetoric that differs from their parties’ common language. Our findings demonstrate the functional use of strategic moral rhetoric in a campaign context and show that unique methods of text network analysis are broadly applicable to the study of campaigns and social movements.

**Significance Statement:**

Of the different persuasion techniques available to candidates on the campaign trail, appeals to moral notions of right and wrong are among the most powerful. Despite this, the fields of political communication and political psychology have yet to empirically investigate and measure the moral language actually used on campaigns. Using a comprehensive data set of digital campaign communications from recent US presidential campaigns, we find that Democratic and Republican candidates consistently use divergent moral language and reveal the extent and manner in which outsider candidates deviate from established rhetorical norms. These findings map our previously uncharted moral-political landscape and suggest the ways in which patterns of moral expression establish rhetorical networks of political division and unity during contentious elections.

## Introduction

At the very heart of elections in a representative democracy lies the art of rhetoric. As Aristotle observed, effective rhetoric can offer political advocates significant electoral influence. Moral rhetoric—emphasizing notions of what is considered morally right or wrong—is among the most powerful and widely used forms of rhetoric. Surprisingly, 25 years into a political era characterized by moral emotion (1–3) it remains unclear exactly how moral rhetoric shapes our electoral landscape. Of particular concern is whether ideologically opposing candidates emphasize different moral values in their rhetoric, thereby entrenching voters in their existing views and exacerbating political polarization (4). Similarly unclear is the extent to which the use of unique moral rhetoric separates candidates from their competition, potentially increasing their persuasive appeal and shaping electoral outcomes (5, 6). However, since campaigns generate vast amounts of textual data and develop nuanced, overlapping vocabularies, empirically mapping the moral language used by competing candidates poses a unique challenge.

Here, we develop a methodology combining natural language processing and network analysis to reveal the extent to which the use of specific moral words connected or differentiated political candidates during recent elections in the United States. Focusing on five moral foundations (7, 8)—care, fairness, loyalty, authority, and sanctity—and leveraging a complete data set of 139,412 tweets published by 39 candidates during the 2016 and 2020 US presidential primaries, we illustrate how moral word choice organizes candidates in rhetorical space. This methodology is then applied to answer two key theoretical questions: (1) To what extent do political candidates within and between party lines naturally converge or diverge based on their use of moral words, and what moral-rhetorical dynamics drive these patterns? (2) To what extent can the use of unique moral rhetoric separate candidates from their competition?

In line with preceding work on elite moral rhetoric, we use moral foundations theory (MFT) (7) to operationalize our analysis. Despite ongoing discussion surrounding its usefulness, MFT offers both methodological and theoretical advantages in the context of our analysis. First, it offers an array of validated methodological tools (9–11) for the extraction of moral language from short texts, facilitating analysis of moral rhetoric in candidates’ rhetoric at scale (an in-depth rationale for our selection of moral foundations dictionary (MFD) can be found in the Supplementary Appendix Section 1.2.1). Second, it allows our paper to build directly on existing work examining moral language in the political public sphere (5, 6, 12, 13). MFT thus provides a helpful methodological and theoretical grounding for our investigation and enables the continuity of academic discourse on the nuanced relationship between moral language and political rhetoric.

Our work differs from prior scholarship on elite moral rhetoric in two main ways. First, we expand the study of rhetorical positioning during elections. By empirically illustrating how candidates are *connected* by shared vocabularies or *differentiated* through the use of language uniquely their own, our approach can reveal not only the moral-rhetorical norms of a given primary but also the extent and manner in which outsider candidates deviate from those norms. Our approach is distinct from those treating the rhetoric of candidates as discrete objects of study (14) and provides important context: candidates argue, debate, and respond *to* one another as part of a connected discourse, and the moral language a candidate chooses to use may depend on the utterances of their peers. Voters may also select candidates based on their proximity to a moral-rhetorical ideal point, making electoral outcomes for a given campaign context-dependent. It is therefore important to understand not just what a candidate says during a campaign, but rather what a candidate says, *given what other candidates are saying*. We argue here that by choosing to use some words and not to use others, competing candidates create a sociolinguistic map which can be reconstructed and analyzed (15–17), making visible the ways in which moral language structures inter- and intra-party dynamics, ideological shifts, and electoral outcomes.

Second, our study comprehensively examines discrete moral values—operationalized here as moral foundations (18)—in campaign rhetoric, bridging the gap between lab-based work on moral foundations and rhetoric in a campaigning context. Previous work on elite moral rhetoric has instead emphasized either discourse from already-elected leaders—analyzing “official” Twitter accounts and congressional floor speeches (19–21)—or the diffusion of moral messages online (12, 22–24). This leaves the precise moral content of candidate messages—especially during primaries when political outcomes are undecided—unclear. Filling this research gap is important: the specific moral foundations used in campaign rhetoric can directly induce or reduce support for candidates (13) and greatly influence the degree to which a liberal or conservative individual endorses a political issue (6). Thus, we asked the following questions: how do patterns of moral expression delineate partisan factions, and when are those boundaries crossed? The present work begins to fill this research gap, mapping this previously uncharted domain and illustrating how the use of moral language connects candidates and parties to form a moral-political landscape.

## Results

**Fig. 1.**
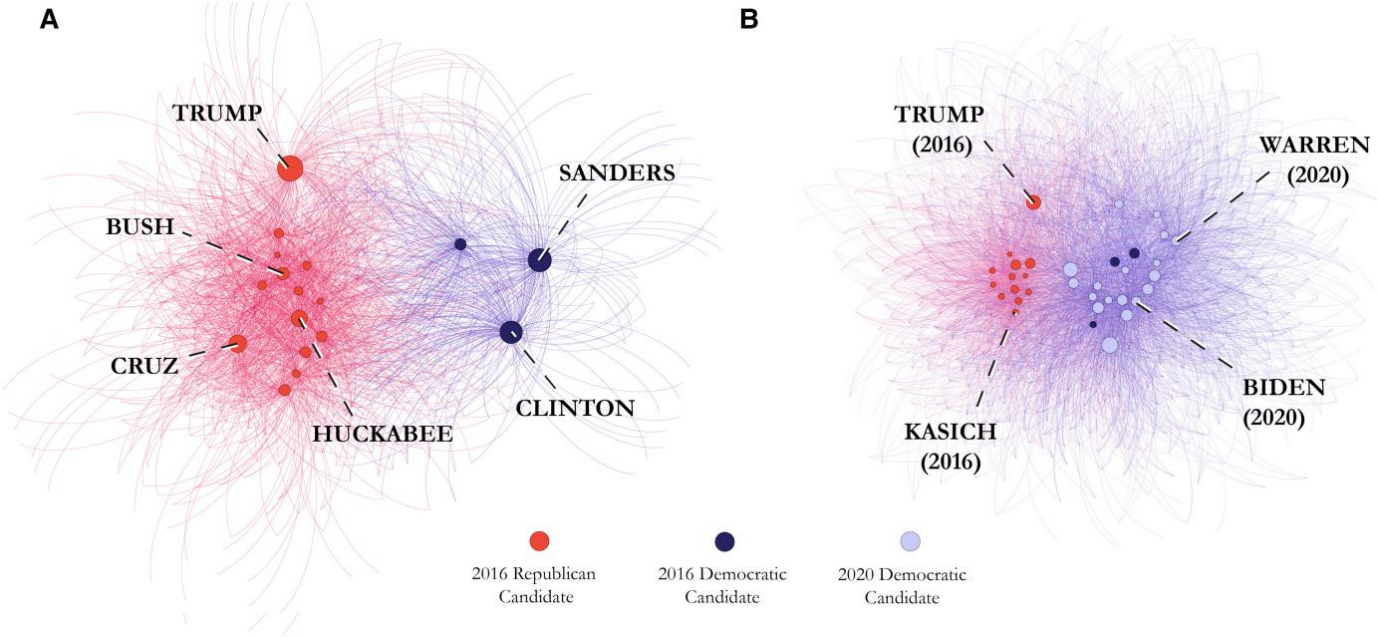
A) Bipartite text network displaying the moral-rhetorical community structure of the 2016 US presidential primaries, based on a frequency analysis of 574 moral terms used by 17 Democratic and Republican candidates on Twitter. Nodes were colored using a Louvain community detection algorithm, which detected two communities reflecting partisan affiliation (Q=.24). Candidates are connected to each other through their use of the same moral words. Word nodes were removed to enhance readability, leaving spatialized candidate positions. Node and label sizes scale with betweenness centrality. Edges are colored by their candidate source node. B) Bipartite text network displaying the moral-rhetorical community structure across both the 2016 and 2020 US presidential primaries, based on a frequency analysis of 574 moral terms used by 34 Democratic and Republican candidates on Twitter (Q=.12). Candidate nodes are connected to each other through their use of the same moral language and colored by partisan affiliation; word nodes have been removed to enhance readability, leaving spatialized candidate positions. Node sizes are scaled by betweenness centrality. Edges colored by source node. Some candidate labels were removed to enhance readability.

First, we determined the extent to which political candidates within and between party lines naturally converged or diverged based on their use of moral words. To achieve this, two text networks were constructed from the data set. One network (see Fig. 1A) was constructed using the moral language generated by the 3 Democratic and 14 Republican candidates who competed during the 2016 election. The second network (see Fig. 1B) was constructed using all 39 candidates from both 2016 and 2020 primaries (14 Republican, 25 Democratic).

Given the asymmetric nature of the data set—which contained two Democratic primaries but only a single Republican primary—the construction of two separate networks served to make use of the full data set while also mitigating concerns which might have been present if conclusions were drawn based solely on either one of the networks individually. The network containing only 2016 candidates, for example, serves to mitigate temporal concerns that would arise when comparing 2016 Republican candidates to 2020 Democratic candidates: namely, that any detected difference in use of moral language would be due to temporal context, and not partisanship. Conversely, the network aggregating both 2016 and 2020 candidates serves to confirm—to the extent that the data is able—that any detected difference in use of moral language between 2016 Democrats and 2016 Republicans is not an isolated occurrence, but rather a single data point in a continued trend of moral-rhetorical divergence over time (see Supplementary Appendix, Section 3, Figs. S6–S11 for full-size figures). In total, the 2016 network contained 516 nodes and 2,018 weighted edges, while the aggregated 2016–2020 network contained 537 nodes and 4,841 weighted edges. The community detection algorithm (resolution parameter=2.19) detected communities of candidate nodes overlapping exactly with partisanship affiliation. The aggregated 2016–2020 network contained 537 nodes and 4,841 weighted edges (see Supplementary Appendix Section 1.6, Table S5 for additional network metrics).

**Fig. 2.**
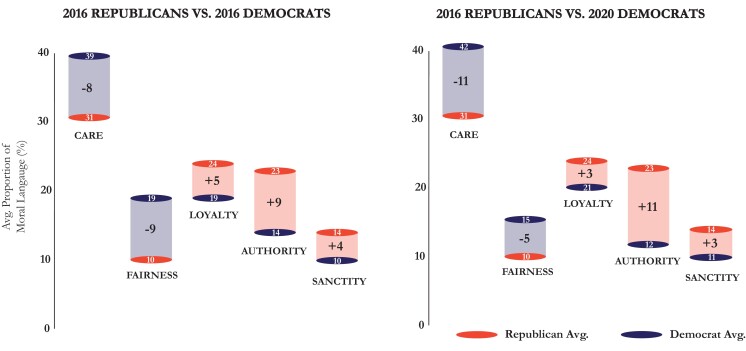
Difference in average proportion of moral foundation use between Democratic and Republican primary candidates on Twitter. Left plot compares 2016 Republican candidates and 2016 Democratic candidates; right plot compares 2016 Republican candidates and 2020 Democratic candidates.

We next investigated the moral-rhetorical dynamics driving this divergence, revealing how and why candidates diverged. Our findings illustrate that the divergence in the network was driven by two related but distinct forces. First, Democratic and Republican candidates were “pushed away” from one another by emphasizing different moral foundations, displayed in Fig. 2. Consistent with the theoretical predictions of MFT (10), Democrats used more care and fairness language, while Republicans used comparatively more loyalty, authority, and sanctity language. The proportion of moral foundation usage between the two groups was significantly different for care (t(36)=5.94,p<0.0001,d=2.35), fairness (t(36)=3.47,p<0.001,d=1.34), authority (t(36)=−7.21,p<0.0001,d=1.8), and sanctity (t(36)=−2.47,p<0.05,d=0.62), but not for loyalty (t(36)=−1.47,p>0.05,d=0.40). Notably, similarities also exist between the parties, with care being the most prominently used foundation by both parties in 2016 and 2020. A comparison of the 2016 and 2020 Democratic candidates finds that they used nearly identical proportions of each moral foundation. This suggests remarkable consistency in use of moral rhetoric by Democratic candidates across recent election cycles.

**Fig. 3.**
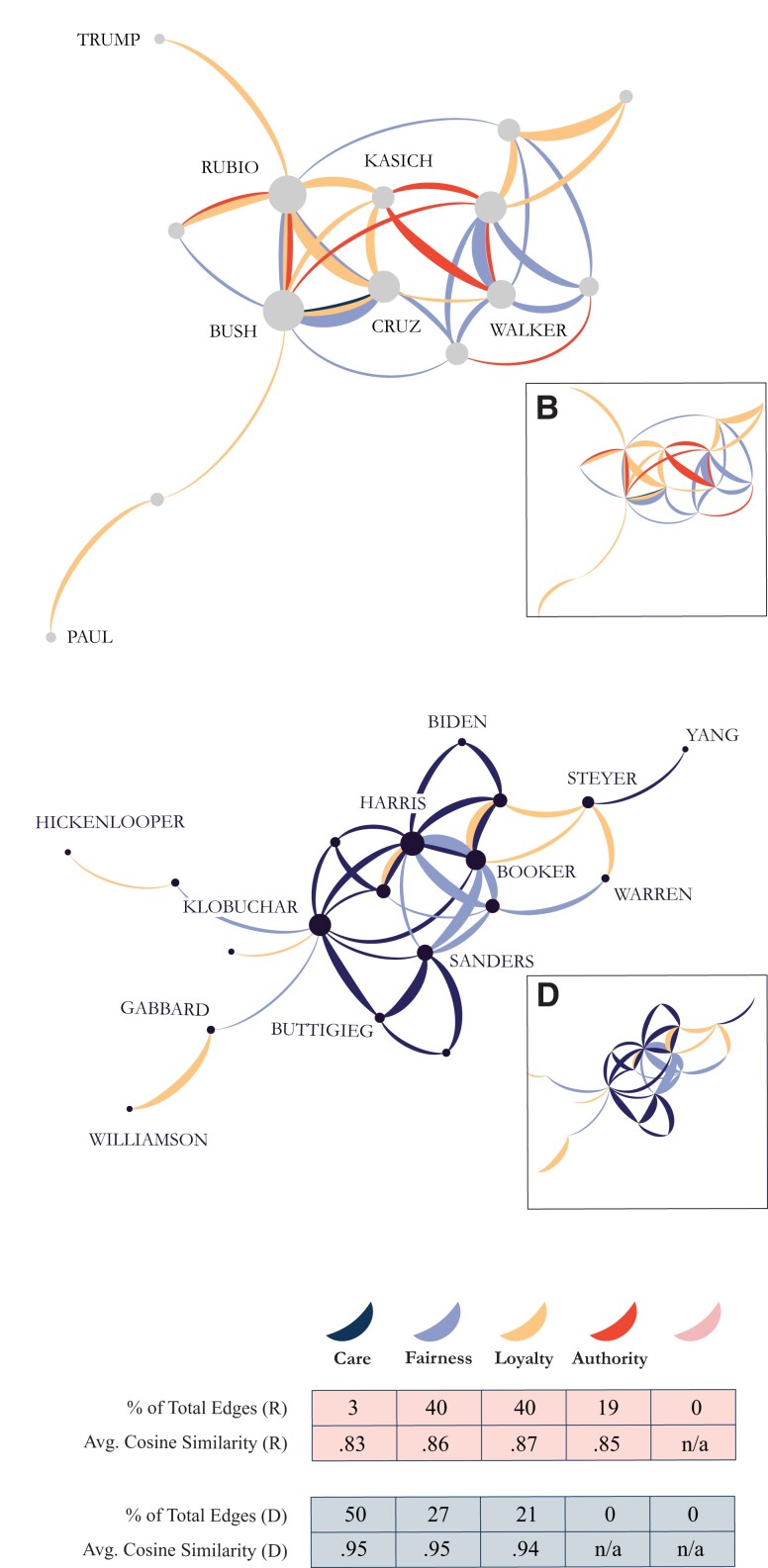
(3a and 3c) Moral foundation similarity networks illustrating how candidates during the 2016 Republican primary (3a) and the 2020 Democratic primary (3c) were connected through their similar use of individual moral foundations. Edge weights index strength of similarity along a given dimension of moral reasoning; edge colors correspond with moral foundation. Node size was scaled with degree; some candidate labels were removed for clarity. (3b and 3d) Network skeletons for each moral similarity network, where nodes and labels have been removed to highlight trends. (3e) Legend and summary statistics for each network. 2020 Democratic candidates show greater cohesion and similarity in their use of moral language than 2016 Republicans, who present a far more fractured network. The most highly endorsed “Democratic” foundations—care and fairness—are also discussed in the most similar ways.

Second, within each party Democratic and Republican candidates were each “pulled toward” each other by discussing their favored moral foundations in highly similar ways. Using cosine similarity networks, we measured the degree to which candidates shared a moral-rhetorical “intra-foundation similarity,” defined here as a similar pattern of word selection and word use, within a given moral foundation. Network analysis allowed us to evaluate the extent to which these patterns of similar moral expression recurred across candidates, revealing the connective nature of specific, intra-foundation vocabularies and their role in the definition of moral-rhetorical norms during a given primary. In other words, Fig. 3 measures and visualizes—across two political parties and two primary elections—the moral foundations which tended to be discussed in the most similar ways.

Fig. 3 reveals that just as Democratic candidates talk about care and fairness more, they also do so in highly similar ways, selecting the same care and fairness words and using them in the same proportions. The average pairwise cosine similarity scores and total edge make-up calculated across all edges in each network reported in Fig. 3e reveal that nearly 80% of the most similar “intra-foundation similarity” relationships between Democratic candidates took place along moral foundations of care and fairness. Likewise, for Republicans, 59% of their most similar “intra-foundation similarity” relationships took place along moral foundations of loyalty and authority. Taken together with the diverging use of moral foundations illustrated in Fig. 2, these findings comprehensively reveal two different dynamics driving the clustering: inter-party moral-rhetorical division, and intra-party moral-rhetorical unity. Critically, it is the combination of both forces which form the diverging clusters displayed in Fig. 1.

**Fig. 4.**
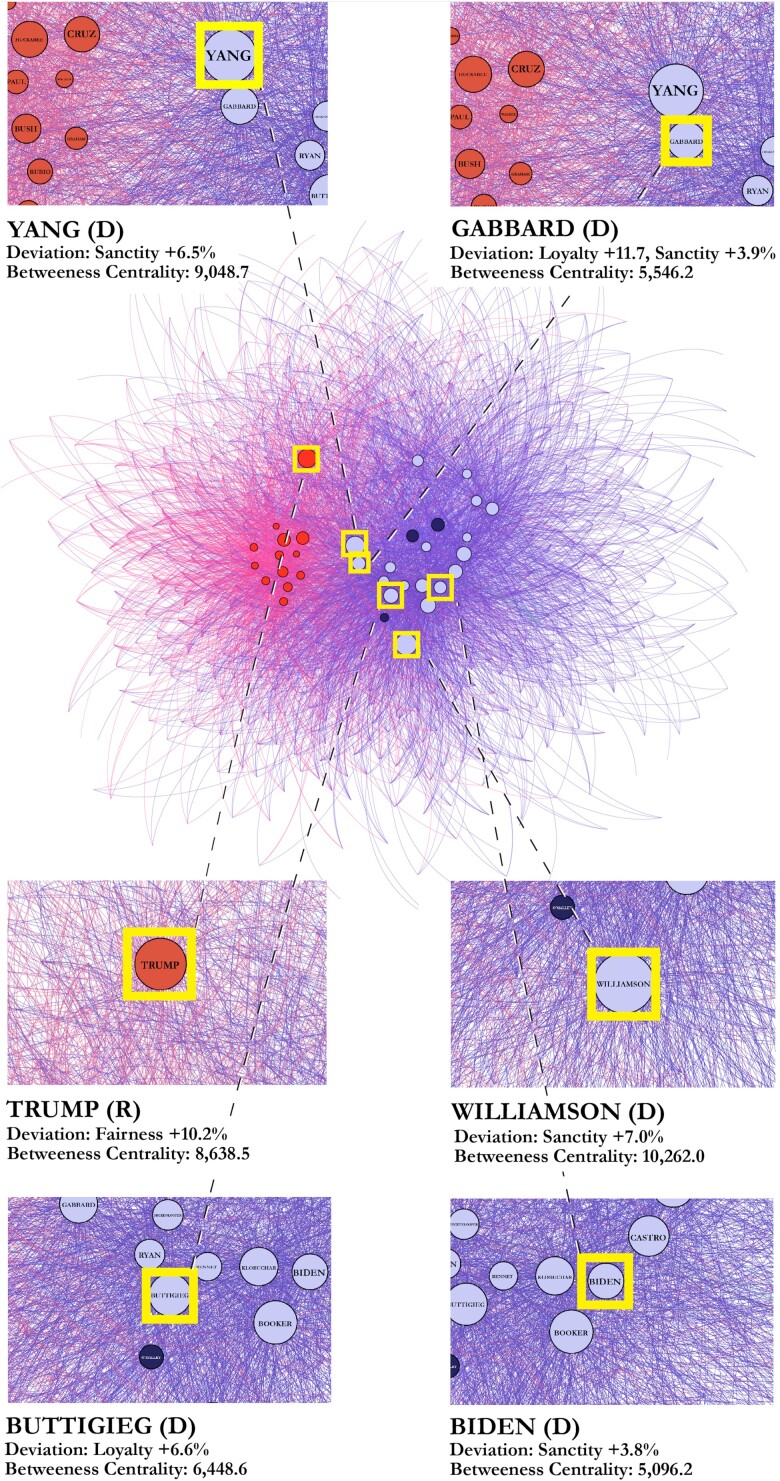
The network position, moral-rhetorical deviation, and betweenness centrality score of six candidates identified as using a significant proportion of moral language associated with a moral foundation endorsed by the opposing party. Their deviation is given in terms of their party average during the election in which they competed (shown in Fig. 2). The average betweenness centrality score for nondeviating candidates was 3579.2.

We next examined our second key question: to what extent can the use of unique moral rhetoric separate candidates from their competition? With an eye toward the strategic incentives that may underly persuasive “moral reframing” tactics (6), instances were identified in which a Democratic candidate used a proportion of loyalty, authority, or sanctity language equal to or greater than the average proportion used by 2016 Republican candidates. Likewise, we identified Republican candidates who used a proportion of care or fairness language equal to or greater than the average proportion used by 2016 Democratic candidates. Their use of moral language within the deviating foundation was then analyzed—along with their network position—to determine the specific moral words driving their moral-rhetorical divergence, and the degree to which their moral-rhetorical divergence co-occurred with network isolation. In total, six significant deviations were identified for further analysis. To reveal the extent to which these moral-rhetorical deviations may have distanced individual candidates from their competitors—their position on the network displayed in Fig. 1B was identified. Fig. 4 illustrates the location of deviating candidates on the network and expresses the foundation in which they deviated. Deviations are expressed in terms of the party average for that primary. Tables with exact deviation data for all 39 individual candidates across all five moral foundations can be found in the Supplementary Appendix Section 2.3, Tables S7 and S8, and are visualized in the Supplementary Appendix Section 3, Figs. S10 and S11.

The extent to which these candidates were isolated in moral-rhetorical space can also be assessed through an analysis of their betweenness centrality scores, a statistic used to determine how much influence a node has over the flow of information in a network. In this context, a large betweenness centrality measure indicates that a candidate favored moral words used by few or no other candidates. As shown by a visual appraisal of the network and validated through a calculation of betweenness centrality statistics, candidates who deviated from norms of moral language use within their parties often acted as network “gatekeepers” to clusters of less-used moral words. Interestingly, their positions within the network layout suggest that these deviations do not necessarily have consistent spatial consequences.

For example, Donald Trump used larger amounts of fairness language than any other Republican candidate in 2016, but this did not result in a network position proximate to other Democratic candidates, who typically use large quantities of this language. Rather, Trump deviated away from both parties by using unique fairness language, unused by Democrats. Table 1 reveals how his fairness vocabulary largely diverged from both parties. By contrast, two Democratic candidates identified on the network—Tulsi Gabbard and Andrew Yang—used high proportions of loyalty and sanctity language and shifted towards the community of Republican candidates. Table 1 reveals that even a list of the top 10 most-used words in each foundation begins to display overlap between the moral language used by Yang and Gabbard and the moral language uniquely favored by 2016 Republicans. Finally, Joe Biden and Pete Buttigieg were able to use significant proportions of sanctity and loyalty language and retain a central network positions amongst their fellow Democrats. They achieved this by emphasizing nonpartisan sanctity and loyalty language (language unused by 2016 Republicans). As Table 1 shows, this novel language was balanced by the use of typical “Democratic” loyalty and sanctity words, which they used in higher proportions than their peers.

**Table 1.** Top 10 most-used words in the category in which a candidate recorded a significant moral-rhetorical deviation, compared with the top 10 terms used by their party and the opposing party. Words highlighted in green indicate a term unique to that candidate; words highlighted in blue indicate a term shared with the opposing party.

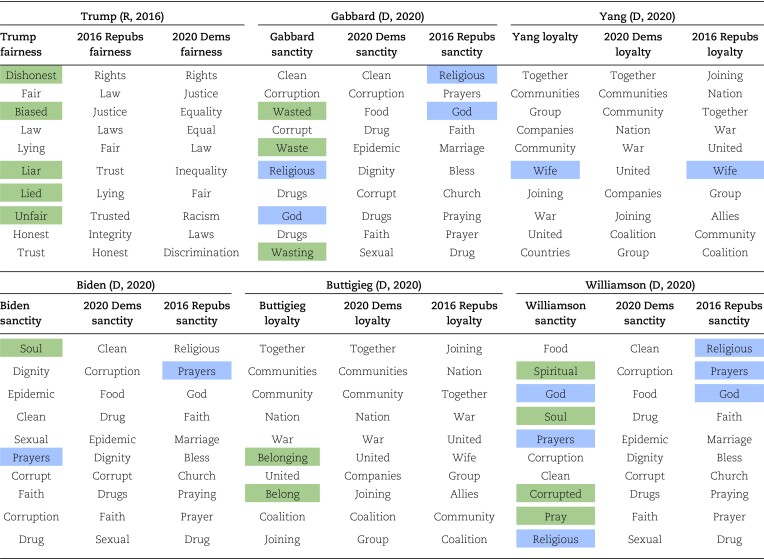

## Discussion

To the best of our knowledge, this is the first study to combine natural language processing and network analysis to map the dynamics of moral rhetoric in online discourse. Our approach uniquely reveals that just as moral convictions play a critical role in constructing the political attitudes of voters, moral language plays a critical role in connecting and differentiating political candidates and political parties during presidential elections in the United States. We find that Democratic and Republican candidates naturally diverged based on their use of moral words and reveal two forces driving this partisan clustering. Firstly, the two parties were “pushed away” from each other by the emphasis of different moral foundations, with Democrats emphasizing careful and just treatment of individuals and Republicans emphasizing in-group loyalty and respect for social hierarchies. Second, candidates within each party were “pulled towards” each other by discussing popular moral values in highly similar ways. Further, our methodological integration of natural language measurement and network analysis allowed for an empirical representation of moral-rhetorical candidate positioning, revealing the way in which use of moral language may isolate a candidate from their peers—as it did Trump in 2016—or insulate a candidate amongst them, as it did Joe Biden in 2020.

These findings confirm that candidates rhetorically diverged along the moral foundations hypothesized by MFT (10), with Democratic candidates using more care and fairness language and Republican candidates using comparatively more loyalty, authority, and sanctity language. Our findings extend existing studies of moral differences between parties (19), suggesting that the partisan nature of primary campaigns (2), the unique incentives faced by political communicators to moralize their rhetoric on digital social networks (12, 23, 24), and trends toward moral-emotional expression in American political discourse more broadly (21, 25) may contribute to greater differences in moral expression between parties than were previously known. Importantly, our findings establish that the moral language used by Democratic and Republican candidates during recent primaries will likely be less persuasive to individuals from opposing parties (6), and may exacerbate political polarization. Notably, a comparison of two Democratic primaries taking place four years apart showed very little variance in the proportion of each moral foundation used, suggesting that these patterns of moral expression may be deeply entrenched norms in Democratic campaigning. The findings indicate that Democratic candidates largely “stuck to their guns” after their general election loss in 2016, despite intense debate surrounding their messaging tactics at the time.

These patterns of moral framing also bear significant implications for digital campaigning and elite political communication on Twitter. Research on diffusion of moralized language in digital social networks has found that the presence of moral-emotional language in political messages is associated with increased diffusion within—but typically not across—partisan communities (12). Our findings might explain why: if political messages from campaigning politicians contain two distinct categories of moral-emotional language, and a political message is more widely shared by individuals who endorse the moral-emotional language it contains, then a polarized network would quickly develop. Under these conditions, the ways in which Democratic and Republicans have tended to express their moral campaign rhetoric might be a contributor to the polarization of online social networks. To the extent that online campaign rhetoric contains moral language which fails to diverge from the standard moral-rhetorical norms of a party, voters may be increasingly likely to be exposed to moral arguments in a manner consistent with digital-political echo chambers.

The moral similarity networks also reveal the strength of moral-rhetorical norms present in a party during a given campaign. During the 2020 Democratic primary, the candidates with the largest network degree—Harris (k=10), Booker (k=9), and Klobuchar (k=9)—overwhelmingly used similar care and fairness language, offering evidence that moral foundations central to a given party are also discussed with the least rhetorical variation and play the largest role in co-creating a “party rhetoric.” The results also map on to existing theories of party ideology during the 2016 Republican primary: the most central candidates (candidates with the highest node degree) in the 2016 Republican moral similarity network—Jeb Bush (k=11), Marco Rubio (k=10), and Ted Cruz (k=8)—have each been theorized as playing important ideological roles during the primary. For example, scholars described Bush as the establishment favorite, Cruz as the ideological favorite, and then, when neither proved acceptable to the other faction, Rubio as the proposed alternative (26). The networks confirmed their ideological centrality along a moral dimension, but their ultimate defeat suggests that perhaps a moral fracture was yet another dimension of the Republican “identity crisis” (27) which has not been often discussed.

The global networks positioning candidates in moral-rhetorical space developed in this research also suggest the relevance of moral language in understanding important political dynamics related to candidate positioning. The results establish that Donald Trump’s status as a political outsider in 2016 (28) corresponded with meaningful differences in his moral-rhetorical style vis-à-vis other candidates, making him a moral-rhetorical outsider as well. His unique use of negatively valanced fairness language pushed him far to the periphery of moral-rhetorical space, away from his own party and the opposition. On the other hand, Gabbard and Yang—Democrats who used increased amounts of loyalty and sanctity language—were identified on the network as having deviated significantly towards the Republicans. Interestingly, they were also the only two candidates who received significant support from the right during the 2020 Democratic primary: Gabbard’s campaign received praise from Steve Bannon, President Trump’s former chief strategist, who was “impressed” with her political talent, and Richard Spencer, the white nationalist leader, plainly stated that he would vote for her (29). Yang also received vocal support from white supremacists (30) and on alt-right message boards (31).

Pete Buttigieg and Joe Biden, Democrats notable for their broad popularity during the primary, also used larger proportions of “loyalty and sanctity” language but did so in a way which allowed them to central network positions within the community of Democratic candidates. While Joe Biden’s framing of the 2020 election as “a battle for the soul of the nation” was a standard appeal to the preservation of spiritual purity (32)—and Pete Buttigieg’s definition of his campaign as an effort to create a sense of “belonging” used classic language associated with group loyalty (33)—2016 Republicans rarely used the same language. In other words, Buttigieg and Biden—even as they used opposing (and more broadly persuasive) moral frameworks—rhetorically insulated themselves amongst their peers by creating new vocabularies of “Democratic” loyalty and sanctity words.

During primary elections—like the ones used in this analysis—candidates are largely incentivized to generate support from in-party voters. However, under these conditions they may still benefit from emphasizing moral foundations favored by the opposing party. In fact, voters of both parties are directly and indirectly influenced by the perceived “electability” of a candidate, and many overtly prefer to nominate candidates who they believe can achieve a measure of bipartisan support in the general election (34). Candidates may thus attempt to appear less offensive to the opposing party by moderating or reframing their rhetoric, even during the primary. However, these candidates walk a delicate rhetorical line: they must attempt to create perceptions of bipartisan appeal among in-party voters without at the same time being perceived as disloyal to their party. By generating *unique* moral vocabularies from “opposing” moral foundations—as did Buttigieg and Biden—our study indicates that candidates may be able to leverage the broadly persuasive power of opposing moral language without being pushed to the periphery of moral-rhetorical space.

This research was limited by the asymmetrical nature of party primaries: depending on factors such as incumbency and political context, primaries do not always contain similar numbers of candidates or take place on the same election years. As a result, this research used 2016 Republican primary rhetoric as a benchmark with which to compare both 2016 and 2020 Democrats. Given the novelty of Twitter’s use as a medium for substantive campaign communication, our analysis also inevitably faced constraints related to the number of historical campaigns we could include in our data set. This could be an important limitation, as our resulting set of outlier candidates—key to deriving conclusions about norm violations—was small. To an extent, interpretation of our findings is also limited by the lab-based nature of existing work on moral language effects—naturalistic field studies examining these effects might capture real-world effects on voters more accurately. Finally, our dictionary tuning process outlined in the Supplementary Appendix Sections 1.3–1.5—which resulted in the removal of a small number of highly generic or contextually problematic moral words (e.g. “president”)—may have also excluded some moral words which unite candidates. Further research could develop an improved methodology which empirically and consistently distinguishes between contextually inappropriate moral terms and legitimately unifying moral language. Despite this, we believe that our approach is an effective way to accurately illustrate both the moral-rhetorical divide between political parties and the moral-rhetorical connections formed by individual candidates within a party.

We identify four directions for future research. Firstly, our approach can be used alongside an analysis of message engagement metrics to quantify the effectiveness of moral vocabularies in generating support across party lines. Secondly, our approach allows for a diachronic mapping of moral rhetoric across election cycles, potentially revealing how outlier rhetoric may be adopted and normalized by candidates and parties during subsequent elections. Thirdly, our approach can be used to examine the extent to which other textual features, such as emotional valence, can also delineate partisan clusters; moral words are unlikely to be the only textual features which differ between Democrats and Republicans. Finally, while existing research has shown that moral appeals also delineate political elites in some European multiparty systems (35), extensions of this work could specifically assess both the extent to which these delineations occur in a campaigning context and the extent to which they can be generalized to different electoral systems.

The patterns of moral expression measured in this study illustrate another dimension along which political discourse in the United States is polarized and bear significant implications for the ways voters engage with campaign messaging, respond to campaign issues, and form opinions about political candidates. This research secondarily contributes by illustrating the promise of networked approaches to the study of politics and political language and demonstrates their effectiveness in answering research questions related to campaign rhetoric and candidate positioning. Mapping the moral language used by political candidates in this way can do much to shed light on the emotional underpinnings of a chaotic and expansive national discourse, revealing the ways in which the democratic process of selecting a president has been shaped by—and may impact upon—the moral convictions of citizens.

## Methods

All tweets published by presidential candidates during the 2016 and 2020 US presidential primaries were collected for analysis (data and Supplementary Appendix can be found here). Presidential primaries were selected for two reasons: first, as opposed to general elections, candidates are generally motivated to attract support from “in-party” voters and may thus engage in increasingly partisan rhetoric. Second, presidential primaries can produce enough candidates—and textual data—to observe and validate meaningful trends on a national scale. Altogether, 39 unique campaigns were assessed, including 24 Democratic campaigns and 15 Republican campaigns spanning the course of the two most recent presidential elections. The complete data set of tweets published by the campaign account of each of the 39 candidates was collected using Twitter’s Academic v2 API endpoints, starting from the day of campaign announcement until the day of campaign suspension for both 2016 and 2020 presidential elections (N=139,401) (see Supplementary Appendix Section 1.1, Tables S1, S9–S11 for corpus statistics). In the case of 2016 primary winners Donald Trump and Hillary Clinton and 2020 primary winner Joe Biden, the date on which they became the presumptive nominee was used as the effective end date of the campaign, as from this point their campaign rhetoric may have shifted as they oriented themselves toward the general election (36).

In order to extract and measure the moral words used in candidate tweets, the MFD 2.0 (37) was implemented using a word-count approach. The MFD 2.0 is an update of the original MFD (10), designed to improve detection of moral signal in short texts such as tweets. The original MFD and MFD 2.0 face several limitations. For example, words are generally confined to a single moral category, when they may have associations with multiple foundations. More advanced dictionaries have been developed to mitigate some of these concerns, such as the eMFD (11), which offers continuous word weightings and crowd-sourced terms, and the DDR (9) which uses distributive models and only a handful of qualitatively selected “seed” terms. However, the benefits of these dictionaries were mitigated in the specific context of the present work, and, in some cases, they presented disadvantages. Most notably, the MFD 2.0 contained specific language of interest to US campaigns not present in alternatives like the eMFD (11) (see Supplementary Appendix Section 1.2.1, Table S2). The performance of the MFD 2.0 on Twitter data has previously been validated and compared to that of other dictionaries (38). For further rationalization of our dictionary selection, along with the supplementary analysis and validation measures we undertook, see Supplementary Appendix Section 1.2.1.

In total, the dictionary contains 2,233 unique words across each of five moral foundations: care, fairness, loyalty, authority, and sanctity. To more precisely facilitate the application of the dictionary to the specific domain and data set relevant for this research, and to address the stated research questions most effectively, the MFD 2.0 was filtered, weighted, and validated through a series of tuning steps and robustness checks (see Supplementary Appendix Sections 1.3–1.5, Figs. S1–S3). In total, the filtering steps can be summarized as follows:

- Out of 1167 distinct moral terms used by the 39 candidates, 510 (43%) were filtered out by the application of a minimum term frequency threshold of 3, meaning that they were never used by any candidate more than 3 times.
- Out of the remaining 657 terms, 83 (14%) were eliminated as “generic” language by a custom *tf-idf* weighting process (see Supplementary Appendix Section 1.4).
- The final custom dictionary consisted of 574 distinct moral terms and be found in Supplementary Appendix Section 2, along with an examination of the effect of the weighting process (including a comparison of raw and tuned dictionary applications: see Supplementary Appendix Section 2, Tables S3, S4, and S6).

### Network construction

The extracted moral language was then used to construct two types of networks: text networks and cosine similarity networks. In the text networks, candidates were connected by mutual use of the same moral word. To achieve this, a bipartite network was constructed where nodes representing candidates were connected by an undirected edge to nodes representing each moral term they used, with edge weights indexing the frequency of a term’s use by a candidate. The network was spatialized with the force-directed Yifan Hu layout (39), shown to be especially effective in visualizing smaller bipartite networks. A Louvain resolution community detection algorithm (40) was then applied to the network to identify clusters of candidates.

The cosine similarity networks were constructed to compare a candidate’s use of each moral foundation (care, fairness, loyalty, authority, and sanctity) with that of their peers, revealing which moral foundations were discussed in the most similar ways. “Similarity” in this case refers to word selection and word use; for example, two candidates using similar authority words at similar frequencies would have a thick “authority edge” connecting them. To construct the cosine similarity networks, the moral language used by each candidate was first filtered into separate documents according to moral foundation. The pairwise cosine similarity was then taken between moral foundations across all candidates, yielding five cosine similarity scores for each candidate pair. These cosine similarities were then re-interpreted as weighted edges, allowing for the construction of a network with every candidate connected to every other candidate by five parallel edges, each representing the similarity of the moral language used from a given foundation.

The edges in the network were then filtered such that the cohesiveness of the final network was maximized, and the total number of edges were minimized. Specifically, the lowest possible filtering threshold was selected which produced a network with no disconnected components and used the fewest total number of edges. This filtering allowed for a visual representation of only the most significant inter-candidate relationships and aided in subsequent analysis by rendering a cleaner network which most clearly displayed the most relevant network structures. Edges were colored by the moral foundation they represented; edges weights were re-scaled to more effectively visualize contrast. Node size was scaled with betweenness centrality. The networks were spatialized with the Yifan Hu layout (39). For a more detailed description of network construction, see Supplementary Appendix Section 1.6, Figs. S4 and S5.

## Supplementary Material

pgad189_Supplementary_Data

## Data Availability

The code and replication materials used for this study are available on OSF at 10.17605/OSF.IO/FZ6KP.

## Previous presentation

These results were previously presented at the International Society of Political Psychology in 2022.

## Preprints

A preprint of this article is published at 10.31219/osf.io/wn5rx.
