# Supplementary material for: Mapping moral language on US presidential primary campaigns reveals rhetorical networks of political division and unity
Source: PNAS Nexus. 2023 Jun 9;2(6):pgad189. doi: 10.1093/pnasnexus/pgad189 (PMC10276347; doi:10.1093/pnasnexus/pgad189)
Supplement: pgad189_Supplementary_Data [file pgad189_supplementary_data.pdf]

Supporting Information for:

Mapping moral language on U.S. presidential primary campaigns reveals rhetorical networks of political division and unity

Kobi Hackenburg, William J. Brady & Manos Tsakiris

correspondence to: kobijohnpotter@gmail.com

This file includes:

Section 1: Methodological Procedure
- 1.1 Corpus Selection
- 1.2 Corpus Construction
- 1.3 Validation
- 1.4 TF-IDF Weighting
- 1.5 Filtering and Lexical Extraction
- 1.6 Network Construction
- 1.7 SI References

Section 2: Materials
- 2.1 Raw & weighted proportions of moral language
- 2.2 Customized moral foundations dictionary
- 2.3 Additional Corpus Statistics

Section 3: Full-size & Supplementary Figures
- 3.1 Word clouds of moral language used by candidates in each moral foundation
- 3.2 Party- and candidate-level distributions of moral foundation use

Figs. S1 to S11
Tables S1 to S11
SI References (1-11)

Section 1: Methodological Procedure

This section will contain additional details regarding all methodological steps undertaken. These include corpus selection, corpus construction, dictionary selection, validation, *tf-idf* weighting, filtering and lexical extraction, and network construction.

1.1 Corpus Selection

Of interest for this research were all tweets published by presidential candidates during the 2016 and 2020 U.S. presidential primaries. In total, 10 Democratic and 17 Republican candidates ran for their respective party nomination in 2016, and 29 Democrats challenged Trump during the 2020 Democratic primary. However, in an effort to filter the dataset to campaigns whose rhetoric was likely more substantial, developed, and relevant, candidates were included only if they participated in at least two official primary debates hosted by their national party (either the DNC or the RNC).

As a result of this filtering, 14 candidates were eliminated from the dataset. In total, 3 Democratic and 15 Republican campaigns were included during the 2016 election cycle, and 21 Democratic campaigns were included for the 2020 cycle. In total, 39 unique campaigns were assessed, including 24 Democratic campaigns and 15 Republican campaigns spanning the course of the two most recent presidential elections.

1.2 Corpus Construction

The complete dataset of tweets published by the campaign account of each of the 39 candidates was collected using Twitter's Academic v2 API endpoints, starting from the day of campaign announcement until the day of campaign suspension for both 2016 and 2020 presidential elections ($N = 139{,}401$). Tweet collection was done in R using the `academictwitteR` package (1). See **Table S1** below for summary statistics:

Primary	Candidates	Avg. Campaign Length (days)	Tweets	Tweets per Campaign per Day
2016 Republican	15	244	40,607	10
2016 Democratic	3	350	15,520	14
2020 Democratic	21	301	83,274	12
**Average**	**13**	**298**	**46,467**	**12**
**Total**	**39**	**-**	**139,412**	**-**

Table S1 Summary statistics for the full corpus of candidate primary tweets, including the average number of tweets per campaign per day.

Tweets for each candidate were then concatenated and pasted into a plain text document. All tweets were cleaned through the removal of hashtags, Twitter handles, emojis, and punctuation; all characters were converted to lowercase (2). At this stage, any candidate who tweeted 1.5 standard deviations less than the average candidate in their party and election cycle was eliminated as an outlier: this resulted in the elimination of 1 Republican and 2 Democratic candidates. All further data wrangling was done in R using the `quanteda` package for textual analysis (3). More detailed information about the dataset, including summary statistics for individual candidates, can be found in **Section 2.**

1.2.1 Dictionary Selection

In order to extract and measure use of moral language in candidate tweets, the Moral Foundations Dictionary (MFD) 2.0 (4) was implemented. There are a number of dictionaries constructed specifically for the measurement of moral language in bodies of text: notable others include the original MFD (5), the DDR MFD (6), and the eMFD (7). The MFD 2.0 faces limitations: terms can only be included in a single moral category, when in reality they might have an affiliation with more than one: for example, “worship”, included in the “sanctity” category, might also have connotations of respect and submission, which might indicate an additional association with the “authority” moral foundation. A more advanced dictionary might allow for a word-to-moral-foundation contribution score (where “worship” might have a “sanctity” contribution score of 0.8, and an “authority” contribution score of 0.2). Another drawback is the nature of the dictionary construction: while still standard practice and considered by many to be the gold standard, the terms in the dictionary were generated qualitatively by a group of academics and psychologists.

This inevitably biases the terms included towards the dialects and epistemic positionality of the researchers, who were certainly not a representative demographic sample of language users. A more advanced dictionary would likely use crowd-sourcing methods for the generation of the terms (7) or distributional methods using massive global corpora and only a handful of qualitatively selected “seed" terms (6).

However, while these dictionaries are, in a general sense, more technically advanced, specific circumstances applied which mitigated their usefulness in the context of the present work. Below are detailed notes explaining why we did not select these dictionary alternatives (the original MFD (Graham et al., 2009), the eMFD (Hopp et al., 2020), and the DDR MFD (Garten et al., 2018), as well as a supplementary analysis that provides further justification for our selection.

Original MFD (Graham et al., 2009)

- The original MFD authors overtly “recommend that researchers use the MFD 2.0” because although “the full-length version has no better or worse construct validity than the shorter variants… the full-length version more fully captures each foundation.”
- Compared to the original MFD, the MFD 2.0 was also designed to mitigate the low moral signal-detection of MFD on short texts, which makes it especially suitable for an application to Twitter.
- Moreover, on a Twitter corpus, Hoover et al. (2020) find that for classification tasks “while, on average, the MFD and MFD2 dictionaries yield comparable performance … MFD 2.0 appears to offer higher precision compared to the original MFD”.

In summary, while the MFD 2.0 has been basically assessed as interchangeable with the original version, most comparisons detect slight improvements when compared to the original, especially when used on short texts such as tweets. We also want to note that since the MFD2.0 contains all of the same moral words as the original MFD (which has been extensively applied to Twitter data) use of the MFD 2.0 connects the present work to past research showing a strong track record of successful detection of moral foundation language in short texts.

The eMFD (Hopp et al., 2020)

While we believe in a general sense that the eMFD (Hopp et al., 2020) presents advantages over previous moral foundations dictionaries, circumstances apply in the present case which mitigate its advantages in the specific context of our paper. We have tried our best to outline these points below.

- It is first worth mentioning that the eMFD was constructed using annotated long-form media articles. Authors of the eMFD note that the eMFD "may be less useful for annotating corpora that are far afield in content or structure from news articles" and that in the future the authors plan to advance the eMFD further by "complementing it with word scores obtained from annotating other corpora, such as tweets". To our knowledge, such applications of the eMFD on Twitter have yet to take place.

Hopp et al. (2020) also raise three main advantages of the eMFD over existing dictionaries: crowd-sourced moral terms, continuous word weightings across moral foundations, and syntactic dependency parsing. We briefly address below why these advantages presented limited utility to us in the context of this paper:

- *Continuous word weightings:* For our analysis we necessarily used cosine similarity scores to generate network edges in the cosine similarity networks. As a result, we necessarily had to sort moral terms into binary, mutually exclusive categories in order to compute valid cosine similarity scores. Moreover, on a conceptual level, literature (e.g. Feinberg & Willer, 2019) on the persuasive / polarizing nature of various moral framings has yet to engage with the intensity of individual word associations with a given moral foundation. In other words, to our knowledge no literature exists which suggests that words *mildly* associated with a moral foundation have an appreciably different effect than words *intensely* related with a given foundation. While this is an interesting question to explore, it exceeds the scope of the present research.
- *Syntactic dependency parsing*: While syntactic dependency parsing is useful for understanding context – references to "groups of moral actors, their intentions, and the targets of their actions" (Hopp et al., 2020) – our work here makes no attempt to analyze specific rhetorical frames or understand the context motivating the use of specific moral words.

- *Crowd-sourced terms:* Crowd Sourced terms (intuitive annotation by a heterogeneous crowd), present an advantage over the MFD 2.0. However, we conducted a supplemental analysis and determined that the words we used in our "customized" MFD2.0 (achieved through application minimum term-frequency threshold of three at the candidate level and *tf-idf* removal of generic language) were by and large also generated by the eMFD's crowdsourcing methods. We find that there is an extremely high degree of overlap, with all foundations except sanctity having from ~70-90% of the same words. Our analysis suggests that the patterns in moral language we measure would almost certainly have a high degree of overlap had we used the eMFD. We also find that the eMFD excludes important words:

Moral Foundation	Proportion of words used in our filtered MFD2.0 also in the eMFD	Words included in our filtered MFD2.0 but *not* in eMFD
Care Virtue	66/74 (90%)	*feed*, condolences, genero*, nurse*, empathy, mercy, comfort*, hug*
Care vice	79/88 (90%)	*punch*, bully*, crying, endanger*, distressed, tribulation, agony, needy, carnage*
Fairness Virtue	22/28 (79%)	*reparations, fairness, parity, karma, repay, retaliate*
Fairness Vice	48/64 (75%)	*dishonest, sexis*, scam*, crook*, hypocri*, unjust, oppression, freeloading, segregate*, misleading, disparity, deception, defrauded, unequal, cheat*, imbalances*
Loyalty Virtue	55/63 (87%)	*sacrifice*, corps, together, allegiance, familiar, enlist, indivisible, cohorts*

Loyalty Vice	6/9 (66%)	*disloyal, traitor, treason*
Authority Virtue	91/108 (84%)	*boss*, captains, servant*, dominat*, worship, admiral, captain, guide, oligarchy, punitive, noble, polite, obey, acquiesce, dean, mentor, matriarch*
Authority Vice	20/25 (80%)	*anarch*, dissidents, overthrow*, uprising, treason,*
Sanctity Virtue	32/67 (48%)	*god, lord, bless*, pray*, sanctity, soul*, christian*, bible, pastor, yogi, decency, mary, holy, pure, catholics, worship, jesus, immunity, noble, nuns, synagogue, christ, enshrined, atone, immune, divinity, purity, marry, wholesome, righteous, angel, dignified, mosque, pristine, temple*
Sanctity Vice	31/54 (58%)	*pandemic, degrad*, disgust*, damn, horrif*, rot*, sin, trash, shit, contamina*, garbage, plague*, alcoholism, sleazy, swear, stain, fester, godless, damning, incest, swore, abhor*

Table S2. Comparison of our tuned MFD 2.0 and the eMFD (Hopp et al., 2020). We find that ~70-90% of terms overlap in all foundations except sanctity. Some words were collapsed on stems for clarity.

Table S2, aside from showing the substantial overlap between the words in the filtered MFD 2.0 and the eMFD, reveals critical instances where the eMFD leaves out words that are important for our context. For example, the sanctity terms *not* included in the eMFD – overtly christian sanctity language, such as *god, lord, bless*, pray*, sanctity, soul*, christian*, bible, pastor, jesus, holy,* etc. – are characteristic of the religious right in the U.S., and are thus critical to include in the context of this work. In other words, our analysis found that the terms included in the MFD 2.0 actually possessed advantages over those in the eMFD for the specific application undertaken in the present research.

The DDR MFD (Garten et al., 2018)

- Hoover et al (2020) find – specifically for Twitter data – that word-count dictionary-based approaches "in some cases largely outperformed DDR in terms of precision".

Many performance differences between DDR and word-count dictionaries are related to classification tasks, not word-count implementations. For example, Garten et al. (2018) found that the lower performance of word count methods in their classification task was driven by Twitter posts "which included no words from any of the MFD dictionaries meaning the classifier could do no better than chance and often seemed to overfit the limited signal available." Since we were not attempting to use a classifier to extract moral tweets, but rather are focused on the specific moral words uttered by candidates, this presents no issue.

1.3 Validation

Performance of a dictionary on a new domain and a new dataset is not guaranteed, making validation essential (8). However, validation for dictionary methods is a challenge: the granularity of their outputs is such that human coders are unable to produce the same measures reliably (9). This means that it is "essentially impossible to derive gold-standard evaluations of dictionaries based on human coding of documents" (8).

Consequently, to initially validate both the functionality of dictionary and the robustness and consistency of the data set, the MFD 2.0 was applied to the raw candidate tweets and the distribution was assessed in terms of its congruence with Zipf's law. Zipf's law states that in a body of naturally occurring language, the most frequently used term will be used twice as frequently as the second-most used term, three times as often as the third-most used term, etc. In other words, in natural language, the term rank-frequency distribution is an inverse relationship (10).

An assessment of the data's congruence with Zipf's law therefore serves two purposes. First, it offers a means of assessing whether the dictionary succeeded in capturing a balanced and holistic selection of naturally occurring moral language: in a corpus of millions of words, and a dictionary containing thousands of words, the relationship predicted by Zipf's law should apply. Second, it serves to assess whether the individual candidate distributions of moral language were consistent

and robust, free from outlier values. For example, if certain candidates used automated social media tools which tweeted the same message repeatedly, this would be reflected in their rank-frequency distribution.

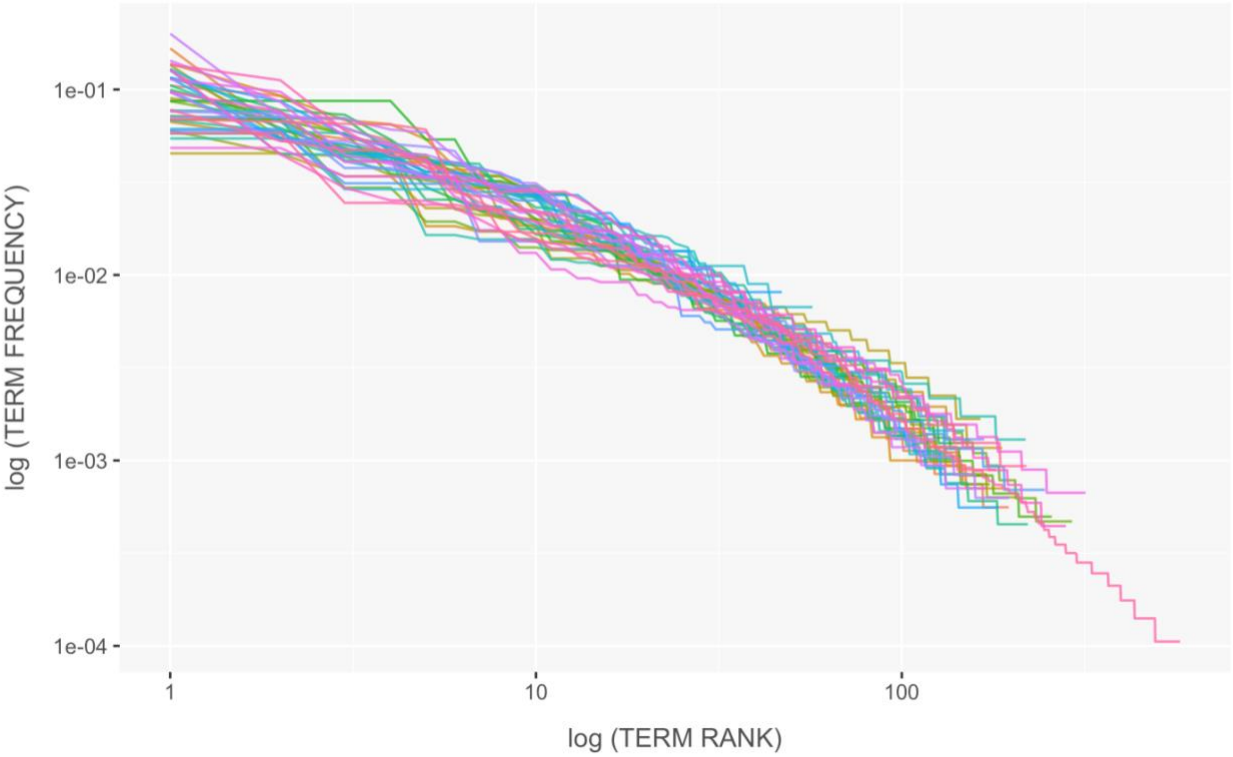

Figure S1 Rank-frequency distribution of the moral terms by candidate. Terms were extracted from over 139,000 tweets from 39 U.S. presidential campaigns. Both axis are scaled logarithmically, making a clear congruence with Zipf's law easily visible across all candidates, 2016-2020.

On a linear scale, Zipf's law takes the form of a power law distribution, but through a log transformation, the relationship becomes negative and linear. The log transformed plot shown in **Figure S1** clearly displays a negative linear relationship, confirming both that the dictionary is complete enough to be working effectively (capturing a representative and "natural" sample of language), and that distributions of moral language used by each candidate were robust and consistent across both political party, election phase, and election year.

1.4 TF-IDF Weighting

To more precisely facilitate the application of the dictionary to the specific domain and data set relevant for this research, and to most effectively address the stated research questions, the MFD

2.0 was filtered and customized through a series of tuning steps. The goal of this tuning process was to remove terms in the dictionary unlikely to be informative about partisan deviations in use of moral language because of their frequent and consistent use across all candidates — in other words, language that is simply "par for the course" in U.S. presidential election campaigning. The removal of this generic language allows for the isolation of moral terms that are used by some candidates, but not others. This aids in more accurately addressing *RQ1*, as it can then be clearly assessed whether any "partisan" moral language appears to be randomly distributed across moral foundations and candidates, or if partisanship seems to correspond with deviations towards particular moral foundations.

In order to identify language used in high proportion by both Republican and Democratic candidates, a global document-feature matrix was created, where both Democratic and Republican candidates were represented by a document containing moral language extracted using the un-altered MFD 2.0. A minimum term frequency threshold was then applied to every document in the matrix, eliminating from each one the terms not used at least three times. This document-feature matrix was then weighted according to a term frequency-inverse document frequency (*tf-idf*) weighting scheme, often used in natural language processing and information science to identify meaningful terms within documents (11). The weighting was applied using the formula

$$w_{i,j} = tf_{i,j} \times log(\frac{N}{df_i})$$

where tf is the number of occurrences of term *i* in document *j*, and df is the number of documents containing term *i*, and *N* is the total number of documents. This weighting scheme has the effect of calculating new frequency scores for each term in the document-feature matrix, where words are down-weighted if they occur with high frequency across many documents and up-weighted if they occur unevenly across documents. *Tf-idf* weighting schemes also value raw frequency: in this context, that means the (often higher-profile) candidates who maintained a campaign over a longer period have more weight in determining what constitutes "Democratic" or "Republican" language; candidates with short, non-communicative campaigns will have their words weighted less. This is useful, as it means that lower tier candidates, while included, will not be able to outweigh the "mainstream" political discourse of a given election cycle.

All terms in the dictionary were then rank-ordered by their newly computed *tf-idf* frequency. Terms ranked near the bottom of this list can be assessed as the least differentiating amongst candidates, and terms near the top are the most differentiating. **Figure S2** shows the distribution of *tf-idf* frequency scores across all moral terms.

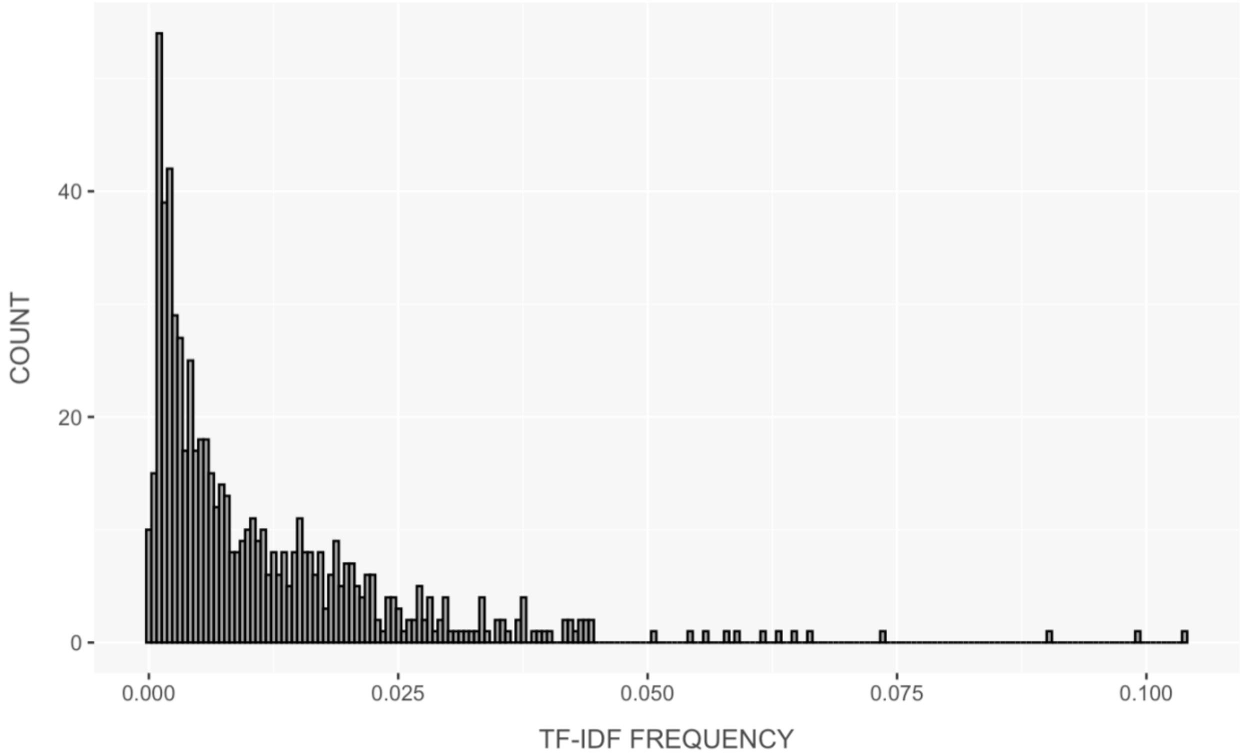

Figure S2 Distribution of 657 moral terms based on their weighted *tf-idf* frequency score. Terms with a lower *tf-idf* frequency score occur often and evenly across all candidates; terms with a higher *tf-idf* frequency score occur less often and more unevenly across candidates.

1.5 Filtering & Lexical Extraction

In order to determine where to set the filtering threshold for "generic" language, Zipf's law was once again implemented. **Figure 2**, used earlier to validate the dictionary, was generated again, this time using the new *tf-idf* frequencies. The result in **Figure S3** shows an intuitive threshold where *tf-idf* term frequency rapidly drops off and falls to 0.

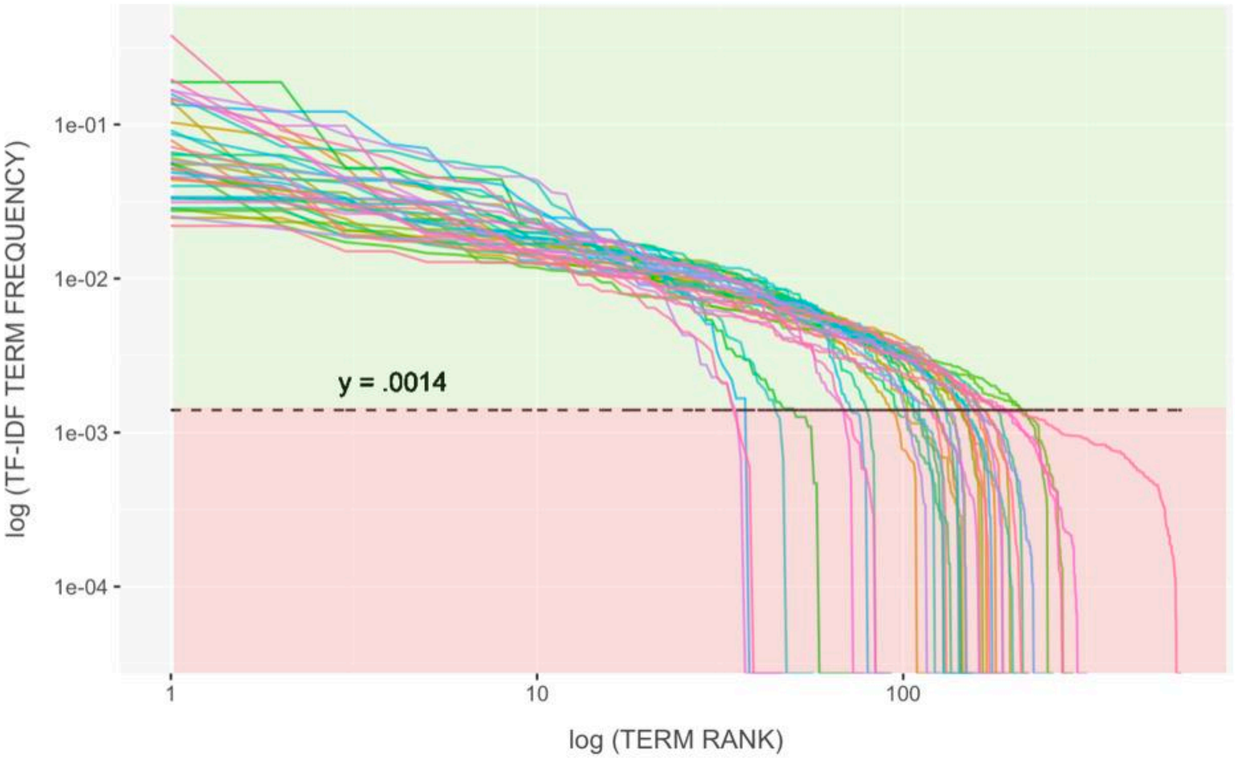

Figure S3 Rank-frequency distribution of moral terms by candidate, weighted by *tf-idf*, and scaled logarithmically. The resulting graph shows a clear threshold where *tf-idf* frequency begins to fall off rapidly across candidates.

A *tf-idf* frequency score of .0014 was intuitively selected as the cutoff point for the terms in the document feature matrix. 83 terms with a tf-idf frequency of less than .0014 — the least informative words — were eliminated. The very least informative of these were "president", "presidential", "family", "love", "country", "leadership", "leaders", "protect", and "fight". The remaining 574 terms — representing not just the moral language used by each candidate, but the *meaningful, non-generic* moral language used by each candidate — were used to construct a new dictionary and were implemented in all subsequent analysis.

Filtering out hyper-frequent, hyper-generic moral language also serves to "tune" the dictionary to the specific context of this research. The MFD 2.0 was not specifically designed to be implemented on a corpus containing only American presidential campaign rhetoric, and as a result there are words in the dictionary that take on less significance. For example, the word "president" is in the dictionary under the "authority" key. Obviously, counting "president" as an indicator for authoritarian moral reasoning is nonsensical in the context of a presidential campaign, where

candidates of all ideologies will use the word frequently. This filtering therefore also serves to remove language that is uninformative for this research context.

During the construction of the aggregated candidate document-feature matrix, iterative tests concluded that as long as candidates were upsampled such that half were Democrat and half were Republican, the exact candidates added to the global document feature matrix (e.g. just 2016 election candidates, both 2016 and 2020 election candidates, primary candidates, both primary and general election candidates) did not have an impact on the moral terms that were ultimately removed during the filtering process. This suggests that the filtering succeeded in eliminating terms that were consistent not just across partisan affiliation but also election year and election phase. See validation **Tables S3 & S4** below:

**2016 Repub & Top 9 2020 Dems**		**All 2016 Candidates**		**All 2016 & 2020 Candidates**	
Term	*TF-IDF Score*	*Term*	*TF-IDF Score*	*Term*	*TF-IDF Score*
president	0	president	0	president	0
together	0	together	0	together	0
safe	0	safe	0	safe	0
family	0	family	0	family	0
presidential	0	presidential	0	presidential	0
love	0	love	0	love	0
country	0	country	0	country	0
leader	0	leader	0	leadership	0
leadership	0	leadership	0	leaders	0
leaders	0	leaders	0	nation	0
nation	0	nation	0	protect	0
protect	0	protect	0	fight	0
fight	0	trust	0	law	0
law	0	fight	0	families	0
attacks	0	law	0		
families	0	attacks	0		
		families	0		

	**Care**	**Fairness**	**Loyalty**	**Authority**	**Sanctity**

**Distributions of terms in the full MFD 2.0 (%)**	24.1	18.0	9.7	21.8	26.3
**Distributions of removed "generic" terms (%)**	25.3	15.6	8.4	22.8	25.3

Table S3. Moral foundations of the 83 moral terms removed as "generic" by the *tf-idf* weighting process, compared with the initial number of terms included in each category of the MFD 2.0. The removed words were distributed evenly across moral foundations, matching the initial distribution of terms. In no case did the filtering alter the overall proportion of moral language used by more than a few percentage points in either direction.

	**Democrats**	**Republicans**
**Tweets containing moral language (before filtering)**	**57,944 / 98,794 (58.7%)**	**19,964 / 40,607 (49.4%)**
**Tweets continuing moral language (after filtering)**	**44,568 / 98,794 (45.1%)**	**13,923 / 40,607 (34.3%)**
**Net change:**	**-13.6%**	**-15.1%**

Table S4. Proportion of tweets containing moral language before and after filtering, showing both the base rates of moral language at the tweet level and that the filtering impacted Democratic and Republican candidates in a balanced way.

In total, the weighting, filtering, and tuning of the dictionary can be summarized through the following steps:

- Out of 1167 distinct moral terms used by the 42 candidates, 510 (43%) were filtered out by the application of a minimum term frequency threshold of 3, meaning that they were never used by any candidate more than 3 times.
- Out of the remaining 657 terms, 83 (14%) were eliminated as generic language by the *tf-idf* weighting process.
- The final custom dictionary consisted of 574 distinct moral terms, and be found in **Section 2.2.** An examination of the effect of the weighting process, including a comparison of pre- and post-weighting proportions of moral language used at the candidate level, can also be found in **Section 2.1.**

1.6 Network Construction

Two different types of networks were constructed for this analysis. One type further addresses *RQ1* & *RQ3*, and aims to reveal both the community structure of partisan moral discourse & the spatial relationships between Democratic and Republican candidates. The second type of network aims to address *RQ2*, and therefore attempts to illustrate how individual candidates within the same party are connected to each other based on their similar use of individual moral foundations. The networks were constructed as follows:

Network Type A: Two-mode network connecting Democratic and Republican candidates to moral language they used on Twitter during their campaigns. These networks were constructed using a combination of R, Gephi and Cortext Manager[1] according to the following steps:

I) The initial incidence matrix M was defined by the number of times moral term i appeared in document of aggregated candidate tweets j.

II) A weighted network was thus constructed such that every time a candidate used a moral word, an edge was drawn between that candidate node and a node representing that moral term. Candidate nodes were never directly linked; their connectedness only occurred through use of the same moral term. Edges in the resulting network were undirected.

III) The network was spatialized with the force-directed Yifan Hu layout (Hu, 2005), which has shown to be especially effective in visualizing smaller bipartite networks.

IV) A Louvain resolution community detection algorithm was then applied to the network to identify clusters of candidates.

Network Type B: A one-mode network connecting candidates to each other through their similar use of individual moral foundations. This type of network was constructed as follows:

[1] https://www.cortext.net/projects/cortext-manager/

I) For each candidate, five documents were created, with one containing the total extracted moral language for each moral foundation. (For example, Biden care, Biden fairness, Biden sanctity, etc.)

II) The pairwise cosine similarity was then calculated for each pair of candidates over each of the five moral foundations subcategory (For example, Biden care and Sanders care, Trump authority & Cruz authority). This process yielded five cosine similarity scores for each combination of two candidates. Cosine similarity was calculated with the vector notation formula:

$$\cos(\theta) = \frac{\mathbf{A} \cdot \mathbf{B}}{\|\mathbf{A}\| \|\mathbf{B}\|}$$

Cosine similarity was selected over other document similarity measures (e.g. Euclidean distance) because it computes similarity based on the proportion of similar words and word frequencies. Other document similarity measures (e.g. Euclidean distance) would have been inappropriately influenced by document length, giving higher similarity scores to candidates who used similar raw quantities of moral language. Given the uneven campaign lengths and tweeting habits across candidates, this was not desirable.

III) The resulting pairwise cosine similarity scores were then re-interpreted as weighted edges and used to construct a perfectly dense network, with each candidate connected to every other candidate by five parallel edges of varying weights (with each edge representing a different moral foundation).

IV) Edges in the network were then filtered such that only edges exceeding a given weight threshold θ were conserved. This threshold was computed based on two criteria: the cohesiveness of the final network and the total number of edges. Specifically, the lowest possible θ was selected which produced a network with no disconnected components using the fewest total number of edges. This filtering also allowed for a visual representation of only the most significant inter-candidate relationships, and aided in subsequent analysis by rendering a cleaner network which most clearly displayed the most relevant network structures.

V) Edges were colored by the moral foundation they represented; edges weights were re-scaled to more effectively visualize contrast. Node size was scaled with betweenness centrality.

VI) The networks were spatialized with the force-directed Yifan Hu layout (Hu, 2005).

To make sure that the cosine similarity scores calculated for the construction of the cosine similarity network were robust, distributions for each vice and valence were calculated to determine that distributions were not being skewed by single outlier terms, thus biasing the cosine similarities. Cosine similarities were found to be extremely robust; only one outlier term, “help”, was found to be an outlier for the 2016 Republican care virtue category; “help” was subsequently removed from both Democratic and Republican corpora. The term distributions used for validation can be found in the preceding **Figure S4**.

These methods of network construction are novel, and constructed specifically for the present research. As a result, further visualization of their construction is helpful. **Figure S5** provides a helpful visual aid and provides easy-to-follow examples showing the construction process for both networks.

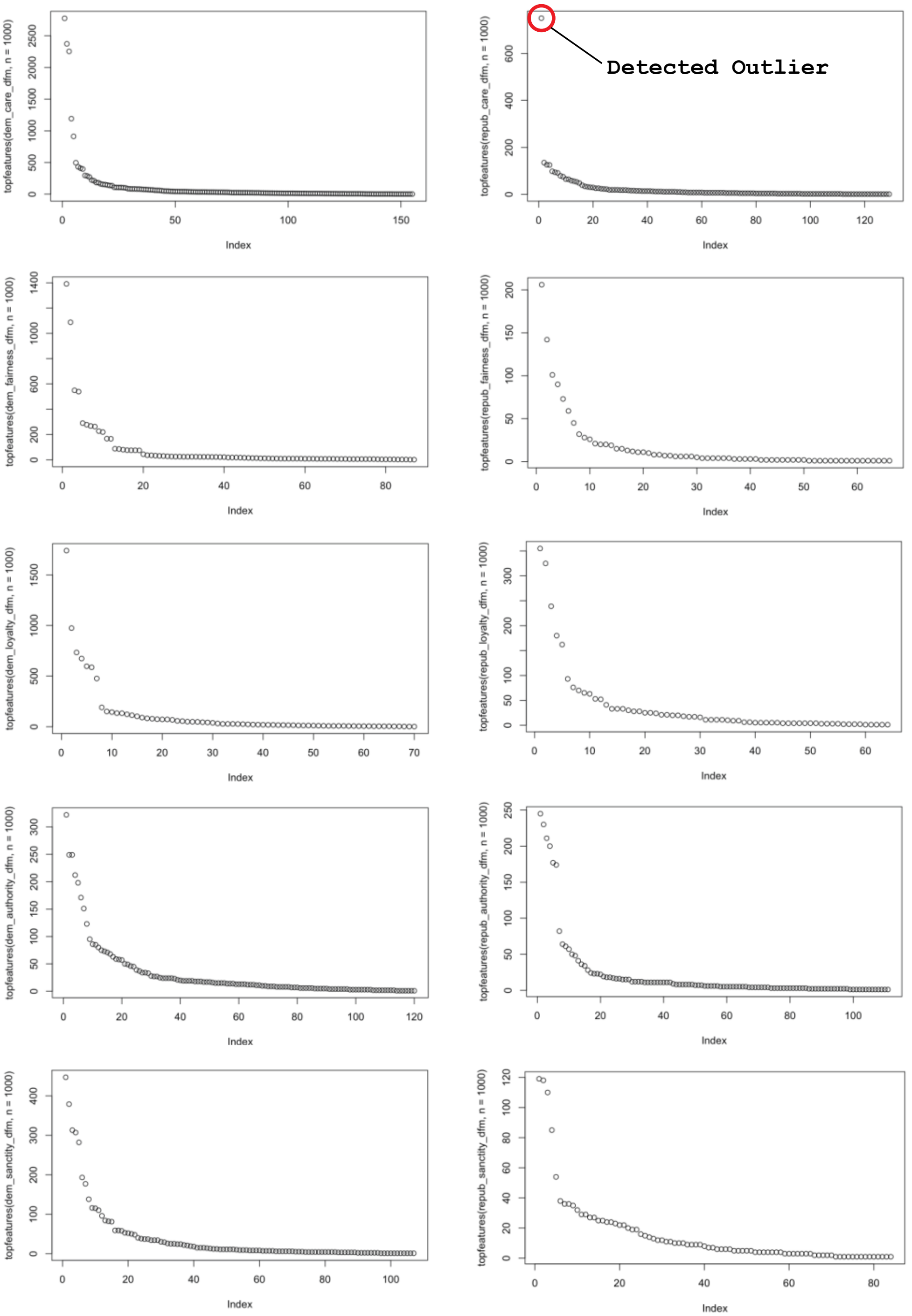

Figure S4 Plots illustrating term distributions used for the calculation of pairwise cosine similarities across moral foundations. A single outlier term “help” was identified and removed from both Democratic and Republican corpora to strengthen the validity of the findings.

Network Type A

Trump: "The media are dishonest liars! No honor!"

Biden: "We must honor our commitment to our communities; unity starts with the familiy."

Step 1: Moral langauge is extracted from candidate tweets

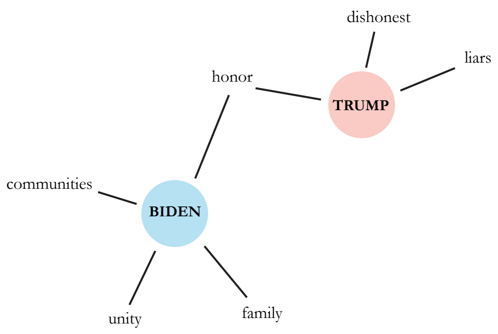

Step 2: Edges are drawn between candidates and moral language they used

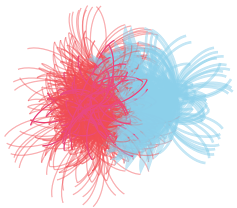

Step 3: Network visualized

Network Type B

Trump: "Our troops will destroy ISIS! ISIS are wounded and vulnerable! Our troops will protect our great nation!"

Biden: "We must help and protect the most vulnerable among us. Together we are a nation united."

Step 1: Moral language extracted and sorted by foundation

Care

	destroy	wounded	vulnerable	help	protect
**Biden**	0	0	1	1	1
**Trump**	1	1	1	0	0

Loyalty

	troops	nation	together	united
**Biden**	0	1	1	1
**Trump**	2	1	0	0

Step 2: Document-feature matrix constructed for each foundation

Trump Care: (1, 1, 1, 0, 0)
Biden Care: (0, 0, 1, 1, 1)

Biden Loyalty: (0, 1, 1, 1)
Trump Loyalty: (2, 1, 0, 0)

Step 3: Each row of the document-feature matrix converted to a vector

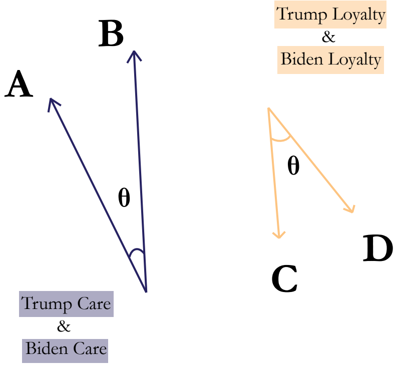

Step 4: Pairwise cosine similarity calculated between each *n*-dimensional vector

Trump Loyalty/Biden Loyalty:

Cosine Similairty = 0.678

Trump Care/ Biden Care:

Cosine Similairty = .543

Step 5: Cosine similarity scores are converted to weighted edges

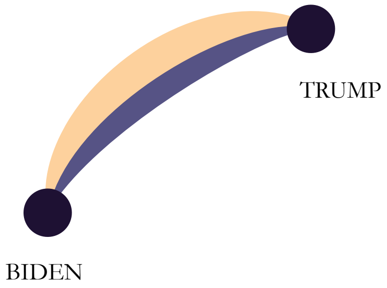

Step 6: Network spatialized, with each candidate connected by parallel edges indexing moral foundation simliarity

Figure S5 The stages of construction for each network type. Illustrations are examples only.

1.6.1 Additional Network Metrics

The following **Table S5** contains additional descriptive metrics for the networks shown in Figure 1 in the main text. These statistics were not used for the analysis, but may aid in interpretation:

	**2016**	**2016+2020**
**Avg. Degree**	7.8	17.4
**Avg. Weighted Degree**	1.34	1.35
**Graph Density**	.015	.03
**Avg. Path Length**	2.73	2.25
**Modularity**	.24	.12

Table S5. Additional network metrics.

1.7 SI References

1. C. Barrie, J. Chun-ting Ho, AcademictwitteR: An R package to access the Twitter Academic Research Product Track v2 API endpoint. *Journal of Open Source Software* (2021).
2. M. J. Denny, A. Spirling, Text Preprocessing for Unsupervised Learning: Why It Matters, When It Misleads, and What to Do about It. *Political Analysis* **26**, 168–189 (2018).
3. K. Benoit, *et al.*, Quanteda: An R package for the quantitative analysis of textual data. *Journal of Open Source Software* (2018).
4. J. Frimer, J. Haidt, J. Graham, M. Dehgani, R. Boghrati, Moral Foundations Dictionaries for Linguistic Analyses, 2.0 (2017).
5. J. Graham, J. Haidt, B. A. Nosek, Liberals and Conservatives Rely on Different Sets of Moral Foundations. *Journal of Personality and Social Psychology* **96**, 1029–1046 (2009).
6. J. Garten, *et al.*, Dictionaries and distributions: Combining expert knowledge and large scale textual data content analysis: Distributed dictionary representation. *Behavior Research Methods* **50**, 344–361 (2018).
7. F. R. Hopp, J. T. Fisher, D. Cornell, R. Huskey, R. Weber, The extended Moral Foundations Dictionary (eMFD): Development and applications of a crowd-sourced approach to extracting moral intuitions from text. *Behavior Research Methods* **53**, 232–246 (2021).
8. J. Grimmer, B. M. Stewart, Text as data: The promise and pitfalls of automatic content analysis methods for political texts. *Political Analysis* **21**, 267–297 (2013).
9. J. Krosnick, P. Lavarkas, N. Kim, "Survey Research" in *Handbook of Research Methods in Social and Personality Psychology*, (1999), pp. 404–443.

10. D. Powers, Applications and Explanations of Zipf's Law. *Methods in Language Processing and Computational Natural Language Learning*, 151–160 (1998).
11. S. Qaiser, *et al.*, "Text Mining: Use of TF-IDF to Examine the Relevance of Words to Documents Text Mining."

Section 2: Materials

2.1 Raw & Weighted dictionary outputs

Table S7 and Table S8 contain all raw and weighted proportions of moral language for each candidate, and **Table S6** is a table visualization of the effect of the filtering process. Raw proportions used the unfiltered MFD 2.0; weighted proportions used the TF-IDF weighted version. *See next page.*

Weighting Effects: 2020 Democratic Primary

Candidate	care.virtue	care.vice	fairness.virtue	fairness.vice	loyalty.virtue	loyalty.vice	authority.virtue	authority.vice	sanctity.virtue	sanctity.vice	Care	Fairness	Loyalty	Authority	Sanctity	Prop. Virtue	Prop Vice
Joe Biden	0.0547843	0.0171287	0.02789937	0.00606128	-0.0181252	0.000245487	-0.13221526	0.001532026	0.02238884	0.01425742	0.071913	0.03396065	-0.0178797	-0.1306832	0.03664626	-0.0452679	0.03922491
Bernie Sanders	0.0636013	-0.0208459	0.02469339	0.00828568	-0.0527948	0.000972233	-0.05050641	0.002421136	0.0074194	-0.00149002	0.0427554	0.03297907	-0.0518226	-0.0480853	0.00592938	-0.0075871	-0.0106569
Elizabeth Warren	0.0656371	-0.0560285	0.04637161	0.01794171	-0.0431839	0.00018707	-0.06360551	0.003042595	0.01115418	0.02619088	0.0096086	0.06431332	-0.0429968	-0.0605629	0.03734506	0.01637348	-0.0086662
Amy Klobuchar	0.0698386	0.005027	0.04141435	0.00618557	-0.0259739	0.000385003	-0.1362816	0.003613206	0.01055388	0.02102552	0.0748656	0.04759992	-0.0255889	-0.1326684	0.0315794	-0.0404487	0.0362363
Pete Buttigieg	0.0668772	0.00746416	0.03276773	0.01026555	-0.0234396	-0.000248676	-0.10729739	0.001006234	0.01219242	0.00625214	0.07434136	0.04303328	-0.0236883	-0.1062912	0.01844456	-0.0188996	0.02473941
Andrew Yang	0.0384987	0.01504784	0.01953667	0.00587359	-0.0113771	0.000730427	-0.1001081	0.000436027	0.02022252	0.01687919	0.05354654	0.02541026	-0.0106467	-0.0996721	0.03710171	-0.0332273	0.03896707
Tom Steyer	0.0613596	-0.0085074	0.0291687	0.03084205	-0.0235539	0.001953513	-0.1164446	0.002855139	0.00693684	0.00814714	0.0528522	0.06001075	-0.0216004	-0.1135895	0.01508398	-0.0425334	0.03529044
Kamala Harris	0.0732851	-0.0077407	0.0525191	0.01066082	-0.0349466	-0.000602093	-0.11972954	0.002623437	0.01515345	0.00969937	0.0655444	0.06317992	-0.0355487	-0.1171061	0.02485282	-0.0137185	0.01464083
Cory Booker	0.0375548	-0.0006865	0.0432379	0.01041425	-0.0386462	0.000342806	-0.07625545	0.001136308	0.01490134	0.0115146	0.0368683	0.05365215	-0.0383034	-0.0751191	0.02641594	-0.0192076	0.02272146
Tulsi Gabbard	-0.0055686	0.01561682	0.01799449	0.00604966	0.0142994	0.00114453	-0.0960384	0.001050572	0.01969006	0.01969908	0.01004822	0.02404415	0.01544393	-0.0949878	0.03938914	-0.049623	0.04356066
Julian Castro	0.0484199	0.0230614	0.03363439	0.00799177	-0.0296045	0.000296674	-0.0969553	0.002788288	0.01021099	0.00524169	0.0714813	0.04162616	-0.0293078	-0.094167	0.01545268	-0.0342945	0.03937982
Beto O'Rourke	0.0439892	0.0311914	0.0413589	0.0138331	-0.0709102	0.000284505	-0.08278859	0.003128928	0.00906392	0.01134058	0.0751806	0.055192	-0.0706257	-0.0796597	0.0204045	-0.0592868	0.05977851
Michael Bloomberg						OUTLIER											
Michael Bennet	0.1054824	0.00988069	0.03221222	0.0110512	-0.057047	0.000492117	-0.12981438	0.004100976	0.01170736	0.01515217	0.11536309	0.04326342	-0.0565549	-0.1257134	0.02685953	-0.0374594	0.04067715
John Delaney	0.0707117	0.0139037	0.02373531	0.00451203	-0.0357184	0.000145618	-0.1020569	0.002042163	0.01723141	0.0069513	0.0846154	0.02824734	-0.0355728	-0.1000147	0.02418271	-0.0260969	0.02755481
Marianne Williamson	-0.0108944	0.01479847	0.02173443	0.00536202	-0.0068721	-0.001128668	-0.04627	0.000939856	0.0232912	0.00507186	0.00390407	0.02709645	-0.0080008	-0.0453301	0.02836306	-0.0190109	0.02504354
Tim Ryan	0.095113	0.0144816	0.01470196	0.00505304	-0.0496318	0.000168988	-0.11008592	0.000844941	0.02113557	0.011289	0.1095946	0.019755	-0.0494628	-0.109241	0.03242457	-0.0287672	0.03183757
Bill de Blasio						OUTLIER											
Kirsten Gillibrand	0.0941919	-0.0108001	0.0579605	0.01062646	-0.0556673	0	-0.13040172	0.001720428	0.0131888	0.01951047	0.0833918	0.06858696	-0.0556673	-0.1286813	0.03269927	-0.0207278	0.02105726
Jay Inslee	0.031725	0.0094637	0.03140927	0.01233844	-0.0148147	0.00027244	-0.1155911	0.002731419	0.03209738	0.00718313	0.0411887	0.04374771	-0.0145423	-0.1128597	0.03928051	-0.0351742	0.03198913
John Hickenlooper	0.0396873	0.0160181	0.0275173	0.00611791	-0.0226419	-0.000189282	-0.0891618	0.000711744	0.0078386	0.00419852	0.0557054	0.03363521	-0.0228312	-0.0884501	0.01203712	-0.0367605	0.02685699
Steve Bullock	0.0606293	-0.0453966	0.0703421	0.00698963	-0.0303956	8.20473E-05	-0.1062708	0.001644067	0.00563814	0.03156398	0.0152327	0.07733173	-0.0303136	-0.1046267	0.03720212	-5.686E-05	-0.0051169
Eric Swalwell	0.0206814	0.0524699	0.027404	0.00468674	-0.0276464	0.000484782	-0.12267277	0.00328608	0.01257057	0.01109782	0.0731513	0.03209074	-0.0271616	-0.1193867	0.02366839	-0.0896632	0.07202532
**Average**	0.052934022	0.003302244	0.035525409	0.009432483	-0.03109308	0.000255294	-0.10139797	0.002096169	0.014634393	0.012488996	**0.05624**	**0.04496**	**-0.0308**	**-0.0993**	**0.02712**	-0.0293972	0.02757519
**Top 9 Average**	0.059048522	-0.005460144	0.035289869	0.011836722	-0.0302268	0.000440641	-0.10027154	0.002074012	0.013435874	0.01249736	0.05358838	0.04712659	-0.0297862	-0.0981975	0.02593323	-0.0227241	0.02138859

Weighting Effects: 2016 Democratic Primary

Candidate	care.virtue	care.vice	fairness.virtue	fairness.vice	loyalty.virtue	loyalty.vice	authority.virtue	authority.vice	sanctity.virtue	sanctity.vice	Care	Fairness	Loyalty	Authority	Sanctity	Prop. Virtue	Prop Vice
Hillary Clinton	0.0600305	0.0180063	0.0546433	0.01484015	-0.0815643	0.000388254	-0.09581776	0.002645659	0.01429844	0.00832523	0.0780368	0.06948345	-0.081176	-0.0931721	0.02262367	-0.0484098	0.04420559
Bernie Sanders	0.0684134	-0.004014	0.02818029	0.01160216	-0.0838484	-0.000121056	-0.0382263	0.002008285	0.01135097	0.0115379	0.0643994	0.03978245	-0.0839695	-0.036218	0.02288887	-0.01413	0.02101329
Martin O'Malley	0.0455339	-0.00514629	0.0416022	0.00400184	-0.0434259	0.000575912	-0.0745141	-0.002181818	0.02803874	0.00445593	0.04038761	0.04560404	-0.04285	-0.0766959	0.03249467	-0.0027652	0.00170557
**Average**	0.0579926	0.00294867	0.041475263	0.01014805	-0.06961287	0.000281037	-0.069519387	0.000824042	0.01789605	0.008106353	**0.06094**	**0.05162**	**-0.0693**	**-0.0687**	**0.026**	-0.0217683	0.02230815

Weighting Effects: 2016 Republican Primary

Candidate	care.virtue	care.vice	fairness.virtue	fairness.vice	loyalty.virtue	loyalty.vice	authority.virtue	authority.vice	sanctity.virtue	sanctity.vice	Care	Fairness	Loyalty	Authority	Sanctity	Prop. Virtue	Prop Vice
Donald Trump	-0.0276412	0.02189608	0.02272779	0.03646521	-0.0370775	0.002896034	-0.0762105	0.02232651	0.02190131	0.01361783	-0.0057451	0.059193	-0.0341815	-0.053884	0.03551914	-0.0963001	0.09720166
Ted Cruz	0.0293633	-0.007429	0.02834479	0.00452434	0.0183001	0.00416087	-0.1312465	0.00786754	0.0385563	0.00545098	0.0219343	0.03286913	0.02246097	-0.123379	0.04400728	-0.016682	0.01457473
John Kasich	-0.0243978	0.0081472	-0.0056197	-0.001320937	0.0595732	0.003507243	-0.0505263	-0.001505036	-0.01107818	0.031555825	-0.0162506	-0.0069406	0.06308044	-0.0520313	0.02047765	-0.0320488	0.0403843
Marco Rubio	0.0750645	0.0175252	0.0310298	0.004768083	-0.0430155	0.002272242	-0.1505541	0.006736146	0.08045514	-0.01368381	0.0925897	0.03579788	-0.0407433	-0.143818	0.06677133	-0.0070202	0.01761786
Ben Carson	0.002381	0.00966592	0.01769955	-0.000367854	0.0160389	0.000906029	-0.1024727	0.00448994	0.0377091	0.00032291	0.01204692	0.0173317	0.01694493	-0.0979828	0.03803201	-0.0286442	0.01501695
Jeb Bush	0.0378028	0.0262159	0.01718006	0.002798393	0.0032823	0.002316656	-0.1447177	0.00868566	0.0360958	0.01090669	0.0640187	0.01997845	0.00559896	-0.136032	0.04700249	-0.0503567	0.0509233
Chris Christie						OUTLIER											
Rand Paul	0.0386291	-0.03196738	0.02777203	0.00214886	-0.0073797	0.000452317	-0.0586487	0.01505258	0.00660189	0.0081111	0.00666172	0.02992089	-0.0069274	-0.0435961	0.01471299	0.00697462	-0.0062025
Carly Fiorina	0.0639865	-0.03002623	0.01827593	0.003840641	0.0601632	0.00227324	-0.1348689	0.00448075	0.01059464	-0.00150165	0.03396027	0.02211657	0.06243644	-0.1303882	0.00909299	0.01815137	-0.0209332
Mike Huckabee	0.015353	-0.0353884	0.03358604	0.00900908	-0.0119959	0.003109955	-0.1019298	0.00708428	0.0593747	0.00518614	-0.0200354	0.04259512	-0.0088859	-0.0948455	0.06456084	-0.005612	-0.0109989
Scott Walker	0.0239674	0.0110321	0.02727784	-0.000472914	-0.0169862	0.003527478	-0.0779805	0.00705495	0.01622638	0.0054075	0.0349995	0.02680493	-0.0134587	-0.0709256	0.02163388	-0.0274951	0.02654911
Lindsey Graham	0.0170618	0.0430414	0.01060364	-0.000128213	0.0246928	0.00226033	-0.114997	0.000859498	0.01128744	0.00757878	0.0601032	0.01047543	0.02695313	-0.1141375	0.01886622	-0.0513513	0.05361179
Bobby Jindal	0.0470341	-0.00889623	0.02235764	0.00575476	-0.0140058	0.00406502	-0.1165993	0.01084006	0.047752	0.00439974	0.03813787	0.0281124	-0.0099408	-0.1057592	0.05215174	-0.0134614	0.01616335
Rick Santorum	0.0235661	-0.02765342	0.01881772	0.00579007	-0.0025017	0.00723758	-0.0868552	0.00651383	0.0477681	0.00796135	-0.0040873	0.02460779	0.00473588	-0.0803414	0.05572945	0.00079502	-0.0001506
George Pataki	0.00834575	0.0370376	0.03196324	-0.000242922	0.0067587	0.00484226	-0.1160631	0.003390122	0.03147743	0.00350889	0.04538335	0.03172032	0.01160096	-0.112673	0.03498632	-0.037518	0.04853595
Rick Perry						OUTLIER											
**Average**	0.022355797	0.005020938	0.023491653	0.007031353	0.0032591	0.003777887	-0.102820547	0.008211674	0.027099174	0.000891315	**0.02738**	**0.03052**	**0.00704**	**-0.0946**	**0.02799**	-0.0266148	0.02493317
**Top 9 Average**	0.025396731	-0.000423673	0.018508614	0.008222652	0.00931833	0.003175661	-0.098457703	0.010998494	0.025211674	-0.002296843	0.02497306	0.02673127	0.01249399	-0.0874592	0.02291483	-0.0200224	0.01967629

Table S6: Visualization showing how the filtering process changed the measured proportion of moral language across candidates and foundations.

Raw Proportion of Moral Language: 2016 Democratic Primary																	
Candidate	care.virtue	care.vice	fairness.virtue	fairness.vice	loyalty.virtue	loyalty.vice	authority.virtue	authority.vice	sanctity.virtue	sanctity.vice	Care	Fairness	Loyalty	Authority	Sanctity	Prop. Virtue	Prop Vice
Hillary Clinton	0.2498239	0.1509744	0.1258511	0.03968068	0.2099084	0.00164358	0.1784456	0.007513501	0.03615872	0.02418408	0.4007983	0.16553178	0.21155198	0.1859591	0.0603428	0.80018772	0.22399624
Bernie Sanders	0.2243103	0.08876449	0.07497001	0.03718513	0.3570572	0.00039984	0.1497401	0.005797681	0.03018792	0.04338265	0.31307479	0.11215514	0.35745704	0.15553778	0.07357057	0.83626553	0.17552979
Martin O'Malley	0.2021818	0.07418182	0.1076364	0.02036364	0.2210909	0.00145455	0.2887273	0.002181818	0.08363636	0.01381818	0.27636362	0.12800004	0.22254545	0.29090912	0.09745454	0.90327276	0.112
**Average**	**0.22543867**	**0.10464024**	**0.10281917**	**0.03240982**	**0.2626855**	**0.00116599**	**0.205637667**	**0.005164333**	**0.049994333**	**0.027128303**	**0.3300789**	**0.13522899**	**0.26385149**	**0.210802**	**0.07712264**	**0.84657534**	**0.17050868**

Raw Proportion of Moral Language: 2020 Democratic Primary																	
Candidate	care.virtue	care.vice	fairness.virtue	fairness.vice	loyalty.virtue	loyalty.vice	authority.virtue	authority.vice	sanctity.virtue	sanctity.vice	Care	Fairness	Loyalty	Authority	Sanctity	Prop. Virtue	Prop Vice
Joe Biden	0.2174379	0.1275683	0.06932285	0.01893872	0.251206	0.00303734	0.2226193	0.005538681	0.06271217	0.04609612	0.3450062	0.08826157	0.25424334	0.22815798	0.10880829	0.82329822	0.20117916
Bernie Sanders	0.2951413	0.1222211	0.08349261	0.03480535	0.257117	0.00321899	0.1289609	0.008449854	0.02846796	0.05230862	0.4173624	0.11829796	0.26033599	0.13741075	0.08077658	0.79317977	0.22100392
Elizabeth Warren	0.2144479	0.2272446	0.08820089	0.0372893	0.2282078	0.000344	0.1375301	0.005985552	0.02187822	0.05304438	0.4416925	0.12549019	0.2285518	0.14351565	0.0749226	0.69026491	0.32390783
Amy Klobuchar	0.2263856	0.1074681	0.08665105	0.0156128	0.2466823	0.00156128	0.2534478	0.007285974	0.03148582	0.04553734	0.3338537	0.10226385	0.24824358	0.26073377	0.07702316	0.84465257	0.17746549
Pete Buttigieg	0.2348873	0.08760246	0.08606557	0.03150615	0.3007172	0.00204918	0.2005635	0.006915984	0.03714139	0.01895492	0.32248976	0.11757172	0.30276638	0.20747948	0.05609631	0.85937496	0.14702869
Andrew Yang	0.2742857	0.07183673	0.04707483	0.01605442	0.2778231	0.0029932	0.2234014	0.001632653	0.04680272	0.04435374	0.34612243	0.06312925	0.2808163	0.22503405	0.09115646	0.86938775	0.13687074
Tom Steyer	0.2558654	0.1164232	0.0894201	0.09871625	0.1646746	0.00575476	0.2246569	0.008410801	0.02567508	0.03098716	0.3722886	0.18813635	0.17042936	0.2330677	0.05666224	0.76029208	0.26029217
Kamala Harris	0.2292484	0.1804041	0.1139701	0.02936473	0.2025004	0.0014537	0.1955226	0.006105539	0.03445268	0.02500363	0.4096525	0.14333483	0.2039541	0.20162814	0.05945631	0.77569418	0.2423317
Cory Booker	0.2180451	0.1510394	0.1163202	0.03162318	0.2494471	0.00088456	0.1508182	0.005307386	0.0495356	0.036046	0.3690845	0.14794338	0.25033166	0.15612559	0.0855816	0.7841662	0.22490053
Tulsi Gabbard	0.2104466	0.09386828	0.04542014	0.01400454	0.3141559	0.00264951	0.2293717	0.008705526	0.05185466	0.05563967	0.30431488	0.05942468	0.31680541	0.23807723	0.10749433	0.851249	0.17486752
Julian Castro	0.1954691	0.1521652	0.09462711	0.03348035	0.249599	0.00080192	0.2323577	0.009021652	0.03428228	0.02085004	0.3476343	0.12810746	0.25040092	0.24137935	0.05513232	0.80633519	0.21631917
Beto O'Rourke	0.1863199	0.1442822	0.1054507	0.03651585	0.3174207	0.0007125	0.1453509	0.009084432	0.0320627	0.03651585	0.3306021	0.14196655	0.3181332	0.15443533	0.06857855	0.7866049	0.22711084
Michael Bloomberg	0.2295831	0.1524843	0.06681896	0.01827527	0.2250143	0.0005711	0.2415762	0.006282125	0.03940605	0.04283267	0.3820674	0.08509423	0.2255854	0.24785833	0.08223872	0.80239861	0.22044547
Michael Bennet	0.2413793	0.08497537	0.06311576	0.02339901	0.3118842	0.00092365	0.221367	0.007697044	0.02463054	0.03109606	0.32635467	0.08651477	0.31280785	0.22906404	0.0557266	0.8623768	0.14809113
John Delaney	0.2373658	0.0936513	0.06901453	0.02226785	0.2736892	0.00268478	0.2163613	0.008843967	0.057012	0.0315856	0.3310171	0.09128238	0.27637398	0.22520527	0.0885976	0.85344283	0.15903349
Marianne Williamson	0.2880926	0.09734763	0.0736456	0.03301354	0.1901806	0.00112867	0.1707111	0.008747178	0.1052483	0.04373589	0.38544023	0.10665914	0.19130927	0.17945828	0.14898419	0.8278782	0.18397291
Tim Ryan	0.3138745	0.1015594	0.03478609	0.0159936	0.267493	0.00039984	0.1823271	0.0019992	0.06077569	0.03478609	0.4154339	0.05077969	0.26789284	0.1843263	0.09556178	0.85925638	0.15473813
Bill de Blasio	0.2485929	0.1444653	0.06660413	0.04221388	0.2579737	0.00375235	0.1697936	0.00750469	0.04315197	0.03095685	0.3930582	0.10881801	0.26172605	0.17729829	0.07410882	0.7861163	0.22889307
Kirsten Gillibrand	0.2407643	0.1987261	0.111465	0.02101911	0.160828	0	0.2073248	0.005095541	0.02770701	0.04426752	0.4394904	0.13248411	0.160828	0.21242034	0.07197453	0.74808911	0.26910827
Jay Inslee	0.1873947	0.1358274	0.08122683	0.03404112	0.2244692	0.00067408	0.2339063	0.009100101	0.08527132	0.02831143	0.3232221	0.11526795	0.22514328	0.2430064	0.11358275	0.81226835	0.20795413
John Hickenlooper	0.2232885	0.1241203	0.0806142	0.01983365	0.2354447	0.00191939	0.2725528	0.004478567	0.03454894	0.02175304	0.3474088	0.10044785	0.23736409	0.27703137	0.05630198	0.84644914	0.17210494
Steve Bullock	0.1415424	0.1612187	0.1142494	0.01110758	0.2392891	0.00095208	0.2748334	0.005077753	0.01142494	0.05426849	0.3027611	0.12535698	0.24024118	0.27991115	0.06569343	0.78133924	0.2326246
Eric Swalwell	0.2282769	0.2371134	0.0736377	0.02135493	0.1745214	0.00368189	0.2091311	0.01546392	0.0353461	0.03681885	0.4653903	0.09499263	0.17820329	0.22459502	0.07216495	0.7209132	0.31443299
**Average**	**0.23209283**	**0.13537447**	**0.080921493**	**0.0287144**	**0.24436254**	**0.00183255**	**0.206281987**	**0.007075397**	**0.042646702**	**0.037641303**	**0.36746731**	**0.10963589**	**0.2461951**	**0.21335738**	**0.080288**	**0.80630556**	**0.21063813**
**Top 9 Average**	**0.24063829**	**0.13242311**	**0.086724244**	**0.03487899**	**0.24204172**	**0.00236633**	**0.193057856**	**0.00618138**	**0.037572404**	**0.03914799**	**0.3730614**	**0.12160323**	**0.24440806**	**0.19923924**	**0.07672039**	**0.80003452**	**0.2149978**

Raw Proportion of Moral Language: 2016 Republican Primary																	
Candidate	care.virtue	care.vice	fairness.virtue	fairness.vice	loyalty.virtue	loyalty.vice	authority.virtue	authority.vice	sanctity.virtue	sanctity.vice	Care	Fairness	Loyalty	Authority	Sanctity	Prop. Virtue	Prop Vice
Donald Trump	0.1629213	0.09550562	0.05176565	0.08868379	0.1729535	0.00842697	0.2728732	0.05216693	0.05497592	0.04895666	0.25842692	0.14044944	0.18138047	0.32504013	0.10393258	0.71548957	0.29373997
Ted Cruz	0.1244186	0.1148256	0.06860465	0.01511628	0.2194767	0.01046512	0.3218023	0.02180233	0.1043605	0.01627907	0.2392442	0.08372093	0.22994182	0.34360463	0.12063957	0.83866275	0.1784884
John Kasich	0.1867794	0.1014604	0.02997694	0.00538048	0.2313605	0.00461184	0.3428132	0.006917756	0.08685626	0.007686395	0.2882398	0.03535742	0.23597234	0.34973096	0.09454266	0.8777863	0.12605687
Marco Rubio	0.1755408	0.1023295	0.01497504	0.00249584	0.2512479	0.00499168	0.3793677	0.004159734	0.04908486	0.02579035	0.2778703	0.01747088	0.25623958	0.38352743	0.07487521	0.8702163	0.13976711
Ben Carson	0.1810585	0.08077994	0.06128134	0.00928505	0.2896936	0.00928505	0.2553389	0.01207057	0.1253482	0.01114206	0.26183844	0.07056639	0.29897865	0.26740947	0.13649026	0.91272054	0.12256267
Jeb Bush	0.1407307	0.1278755	0.0338295	0.0067659	0.2050068	0.00405954	0.394452	0.01894452	0.0744249	0.02097429	0.2686062	0.0405954	0.20906634	0.41339652	0.09539919	0.8484439	0.17861975
Chris Christie	0.1608696	0.1217391	0.06086957	0.00434783	0.2347826	0	0.2434783	0	0.09130435	0.1043478	0.2826087	0.0652174	0.2347826	0.2434783	0.19565215	0.79130442	0.23043473
Rand Paul	0.1820491	0.1003387	0.09229467	0.01397121	0.2464014	0.00677392	0.2548688	0.05165114	0.03175275	0.03302286	0.2823878	0.10626588	0.25317532	0.30651994	0.06477561	0.80736672	0.20575783
Carly Fiorina	0.2053292	0.09404389	0.05015674	0.00940439	0.3040752	0.01097179	0.2805643	0.01097179	0.03134796	0.01253918	0.29937309	0.05956113	0.31504699	0.29153609	0.04388714	0.8714734	0.13793104
Mike Huckabee	0.1405325	0.1849112	0.07988166	0.02810651	0.1434911	0.00961539	0.2588757	0.02366864	0.1442308	0.02662722	0.3254437	0.10798817	0.15310649	0.28254434	0.17085802	0.76701176	0.27292896
Scott Walker	0.1383855	0.1136738	0.06919275	0.00988468	0.1911038	0.00823723	0.416804	0.01647446	0.03789127	0.01812191	0.2520593	0.07907743	0.19934103	0.43327846	0.05601318	0.85337732	0.16639208
Lindsey Graham	0.1154434	0.2385321	0.03287462	0.00840979	0.2568807	0.01223242	0.2775229	0.005351682	0.04357798	0.02140673	0.3539755	0.04128441	0.26911312	0.28287458	0.06498471	0.7262996	0.28593272
Bobby Jindal	0.1952462	0.1086587	0.05602716	0.02037351	0.1375212	0.01018676	0.3089983	0.02716469	0.1375212	0.01697793	0.3039049	0.07640067	0.14770796	0.33616299	0.15449913	0.83531406	0.18336159
Rick Santorum	0.1252144	0.1252144	0.04459691	0.01372213	0.329331	0.01715266	0.2161235	0.01543739	0.1132075	0.01886792	0.2504288	0.05831904	0.34648366	0.23156089	0.13207542	0.82847331	0.1903945
George Pataki	0.08914729	0.1802326	0.07945736	0.00581395	0.1937984	0.01744186	0.3333333	0.007751938	0.09108527	0.02713178	0.26937989	0.08527131	0.21124026	0.34108524	0.11821705	0.78682162	0.23837213
Rick Perry	0.1681416	0.08849558	0.01769912	0	0.2300885	0.00884956	0.3451327	0.02654867	0.1327434	0.02654867	0.25663718	0.01769912	0.23893806	0.37168137	0.15929207	0.89380532	0.15044248
**Average**	**0.15573801**	**0.12366354**	**0.05271773**	**0.01511008**	**0.22732581**	**0.00895636**	**0.306396819**	**0.01881764**	**0.08435707**	**0.027276302**	**0.27940155**	**0.06782781**	**0.23628217**	**0.32521446**	**0.11163337**	**0.82653543**	**0.19382393**
**Top 9 Average**	**0.16885524**	**0.10432203**	**0.051528233**	**0.01727231**	**0.23944424**	**0.00662066**	**0.305062078**	**0.019853863**	**0.072161744**	**0.031193185**	**0.27317727**	**0.06880054**	**0.2460649**	**0.32491594**	**0.10335493**	**0.83705154**	**0.17926204**

Raw Proportion of Moral Language: 2016 & 2020 General Election																	
Candidate	care.virtue	care.vice	fairness.virtue	fairness.vice	loyalty.virtue	loyalty.vice	authority.virtue	authority.vice	sanctity.virtue	sanctity.vice	Care	Fairness	Loyalty	Authority	Sanctity	Prop. Virtue	Prop Vice
Hillary Clinton 2016	0.2498239	0.1509744	0.1258511	0.03968068	0.2099084	0.00164358	0.1784456	0.007513501	0.03615872	0.02418408	0.4007983	0.16553178	0.21155198	0.1859591	0.0603428	0.80018772	0.22399624
Donald Trump 2016	0.1226081	0.1119773	0.04181432	0.2445074	0.1736357	0.00708717	0.2161588	0.02267895	0.02976612	0.03827073	0.2345854	0.28632172	0.18072287	0.23883775	0.06803685	0.58398304	0.42452155
Joe Biden 2020	0.2106653	0.08975713	0.0657339	0.01663147	0.2521119	0.00184794	0.2552798	0.01187962	0.05491024	0.05517423	0.30042243	0.08236537	0.25395984	0.26715942	0.11008447	0.83870114	0.17529039
Donald Trump 2020	0.1637329	0.09513831	0.05965353	0.04875664	0.1739313	0.00922045	0.3023191	0.04316848	0.04708019	0.07166806	0.25887121	0.10841017	0.18315175	0.34548758	0.11874825	0.74671702	0.26795194
**Average**	**0.18670755**	**0.11196179**	**0.073263213**	**0.08739405**	**0.20239683**	**0.00494979**	**0.238050825**	**0.021310138**	**0.041978818**	**0.047324275**	**0.29866934**	**0.16065726**	**0.20734661**	**0.25936096**	**0.08930309**	**0.74239723**	**0.27294003**
**Dem. Average**	**0.2302446**	**0.12036577**	**0.0957925**	**0.02815608**	**0.23101015**	**0.00174576**	**0.2168627**	**0.009696561**	**0.04553448**	**0.039679155**	**0.35061037**	**0.12394858**	**0.23275591**	**0.22655926**	**0.08521364**	**0.81944443**	**0.19964332**
**Repub. Average**	**0.1431705**	**0.10355781**	**0.050733925**	**0.14663202**	**0.1737835**	**0.00815381**	**0.25923895**	**0.032923715**	**0.038423155**	**0.054969395**	**0.24672831**	**0.19736595**	**0.18193731**	**0.29216267**	**0.09339255**	**0.66535003**	**0.34623675**

Table S7: Raw proportions of moral language

Weighted Proportion of Moral Language: 2016 Democratic Primary

Candidate	care.virtue	care.vice	fairness.virtue	fairness.vice	loyalty.virtue	loyalty.vice	authority.virtue	authority.vice	sanctity.virtue	sanctity.vice	Care	Fairness	Loyalty	Authority	Sanctity	Prop. Virtue	Prop Vice
Hillary Clinton	0.309854	0.168981	0.1804944	0.054521	0.1283441	0.002032	0.08262784	0.01015916	0.05045716	0.03250931	0.478835	0.235015	0.130376	0.092787	0.082966	0.751778	0.268202
Bernie Sanders	0.292724	0.08475	0.1031503	0.048787	0.2732088	0.000279	0.1115138	0.007805966	0.04153889	0.05492055	0.377474	0.151938	0.273488	0.11932	0.096459	0.822135	0.196543
Martin O'Malley	0.247716	0.069036	0.1492386	0.024365	0.177665	0.00203	0.2142132	0	0.1116751	0.01827411	0.316751	0.173604	0.179695	0.214213	0.129949	0.900508	0.113706
**Average**	**0.283431**	**0.107589**	**0.144294433**	**0.042558**	**0.19307263**	**0.001447**	**0.13611828**	**0.005988375**	**0.06789038**	**0.035234657**	**0.39102**	**0.186852**	**0.19452**	**0.142107**	**0.103125**	**0.824807**	**0.192817**

Weighted Proportion of Moral Language: 2020 Democratic Primary

Candidate	care.virtue	care.vice	fairness.virtue	fairness.vice	loyalty.virtue	loyalty.vice	authority.virtue	authority.vice	sanctity.virtue	sanctity.vice	Care	Fairness	Loyalty	Authority	Sanctity	Prop. Virtue	Prop Vice
Joe Biden	0.272222	0.144697	0.09722222	0.025	0.2330808	0.003283	0.09040404	0.007070707	0.08510101	0.06035354	0.416919	0.122222	0.236364	0.097475	0.145455	0.77803	0.240404
Bernie Sanders	0.358743	0.101375	0.108186	0.043091	0.2043222	0.004191	0.07845449	0.01087099	0.03588736	0.0508186	0.460118	0.151277	0.208513	0.089325	0.086706	0.785593	0.210347
Elizabeth Warren	0.280085	0.171216	0.1345725	0.055231	0.1850239	0.000531	0.07392459	0.009028147	0.0330324	0.07923526	0.451301	0.189804	0.185555	0.082953	0.112268	0.706638	0.315242
Amy Klobuchar	0.296224	0.112495	0.1280654	0.021798	0.2207084	0.001946	0.1171662	0.01089918	0.0420397	0.06656286	0.408719	0.149864	0.222655	0.128065	0.108603	0.804204	0.213702
Pete Buttigieg	0.301765	0.095067	0.1188333	0.041772	0.2772776	0.001801	0.09326611	0.007922218	0.04933381	0.02520706	0.396831	0.160605	0.279078	0.101188	0.074541	0.840475	0.171768
Andrew Yang	0.312784	0.086885	0.0666115	0.021928	0.266446	0.003724	0.1232933	0.00206868	0.06702524	0.06123293	0.399669	0.08854	0.27017	0.125362	0.128258	0.83616	0.175838
Tom Steyer	0.317225	0.107916	0.1185888	0.129558	0.1411207	0.007708	0.1082123	0.01126594	0.03261192	0.0391343	0.425141	0.248147	0.148829	0.119478	0.071746	0.717759	0.295583
Kamala Harris	0.302534	0.172663	0.1664892	0.040026	0.1675538	0.000852	0.07579306	0.008728976	0.04960613	0.034703	0.475197	0.206515	0.168405	0.084522	0.084309	0.761976	0.256973
Cory Booker	0.2556	0.150353	0.1595581	0.042037	0.2108009	0.001227	0.07456275	0.006443694	0.06443694	0.0475606	0.405953	0.201596	0.212028	0.081006	0.111998	0.764959	0.247622
Tulsi Gabbard	0.204878	0.109485	0.06341463	0.020054	0.3284553	0.003794	0.1333333	0.009756098	0.07154472	0.07533875	0.314363	0.083469	0.332249	0.143089	0.146883	0.801626	0.218428
Julian Castro	0.243889	0.175227	0.1282615	0.041472	0.2199945	0.001099	0.1354024	0.01180994	0.04449327	0.02609173	0.419116	0.169734	0.221093	0.147212	0.070585	0.772041	0.255699
Beto O'Rourke	0.230309	0.175474	0.1468096	0.050349	0.2465105	0.000997	0.06256231	0.01221336	0.04112662	0.04785643	0.405783	0.197159	0.247508	0.074776	0.088983	0.727318	0.286889
Michael Bloomberg	OUTLIER																
Michael Bennet	0.346862	0.094856	0.09532798	0.03445	0.2548372	0.001416	0.09155262	0.01179802	0.0363379	0.04624823	0.441718	0.129778	0.256253	0.103351	0.082586	0.824917	0.188768
John Delaney	0.308078	0.107555	0.09274984	0.02678	0.2379708	0.00283	0.1143044	0.01088613	0.07424341	0.0385369	0.415633	0.11953	0.240801	0.125191	0.11278	0.827346	0.186588
Marianne Williamson	0.277198	0.112146	0.09538003	0.038376	0.1833085	0	0.1244411	0.009687034	0.1285395	0.04880775	0.389344	0.133756	0.183309	0.134128	0.177347	0.808867	0.209016
Tim Ryan	0.408988	0.116041	0.04948805	0.021047	0.2178612	0.000569	0.07224118	0.002844141	0.08191126	0.04607509	0.525029	0.070535	0.21843	0.075085	0.127986	0.830489	0.186576
Bill de Blasio	OUTLIER																
Kirsten Gillibrand	0.334956	0.187926	0.1694255	0.031646	0.1051607	0	0.07692308	0.006815969	0.04089581	0.06377799	0.522882	0.201071	0.105161	0.083739	0.104674	0.727361	0.290166
Jay Inslee	0.21912	0.145291	0.1126361	0.04638	0.2096545	0.000947	0.1183152	0.01183152	0.1173687	0.03549456	0.364411	0.159016	0.210601	0.130147	0.152863	0.777094	0.239943
John Hickenlooper	0.262976	0.140138	0.1081315	0.025952	0.2128028	0.00173	0.183391	0.005190311	0.04238754	0.02595156	0.403114	0.134083	0.214533	0.188581	0.068339	0.809689	0.198962
Steve Bullock	0.202172	0.115822	0.1845915	0.018097	0.2088935	0.001034	0.1685626	0.00672182	0.01706308	0.08583247	0.317994	0.202689	0.209928	0.175284	0.102896	0.781282	0.227508
Eric Swalwell	0.248958	0.289583	0.1010417	0.026042	0.146875	0.004167	0.08645833	0.01875	0.04791667	0.04791667	0.538542	0.127083	0.151042	0.105208	0.095833	0.63125	0.386458
**Average**	**0.285027**	**0.138677**	**0.116446902**	**0.038147**	**0.21326947**	**0.002088**	**0.104884017**	**0.009171565**	**0.05728109**	**0.050130299**	**0.423704**	**0.154594**	**0.215357**	**0.114056**	**0.107411**	**0.776908**	**0.238213**
**Top 9 Average**	**0.299687**	**0.126963**	**0.122014113**	**0.046716**	**0.21181492**	**0.002807**	**0.092786316**	**0.008255392**	**0.05100828**	**0.05164535**	**0.42665**	**0.16873**	**0.214622**	**0.101042**	**0.102654**	**0.77731**	**0.236386**

Weighted Proportion of Moral Language: 2016 Republican Primary

Candidate	care.virtue	care.vice	fairness.virtue	fairness.vice	loyalty.virtue	loyalty.vice	authority.virtue	authority.vice	sanctity.virtue	sanctity.vice	Care	Fairness	Loyalty	Authority	Sanctity	Prop. Virtue	Prop Vice
Donald Trump	0.13528	0.117402	0.07449344	0.125149	0.135876	0.011323	0.1966627	0.07449344	0.07687723	0.06257449	0.252682	0.199642	0.147199	0.271156	0.139452	0.619189	0.390942
Ted Cruz	0.153782	0.107397	0.09694944	0.019641	0.2377768	0.014626	0.1905558	0.02966987	0.1429168	0.02173005	0.261179	0.11659	0.252403	0.220226	0.164647	0.821981	0.193063
John Kasich	0.162382	0.109608	0.02435724	0.00406	0.2909337	0.008119	0.2922869	0.00541272	0.07577808	0.03924222	0.271989	0.028417	0.299053	0.2977	0.11502	0.845738	0.166441
Marco Rubio	0.250605	0.119855	0.04600484	0.007264	0.2082324	0.007264	0.2288136	0.01089588	0.12954	0.01210654	0.37046	0.053269	0.215496	0.239709	0.141647	0.863196	0.157385
Ben Carson	0.18344	0.090446	0.07898089	0.008917	0.3057325	0.010191	0.1528662	0.01656051	0.1630573	0.01146497	0.273885	0.087898	0.315924	0.169427	0.174522	0.884076	0.13758
Jeb Bush	0.178534	0.154091	0.05100956	0.009564	0.2082891	0.006376	0.2497343	0.02763018	0.1105207	0.03188098	0.332625	0.060574	0.214665	0.277364	0.142402	0.798087	0.229543
Chris Christie	OUTLIER																
Rand Paul	0.220678	0.068371	0.1200667	0.01612	0.2390217	0.007226	0.1962201	0.06670372	0.03835464	0.04113396	0.28905	0.136187	0.246248	0.262924	0.079489	0.814341	0.199555
Carly Fiorina	0.269316	0.064018	0.06843267	0.013245	0.3642384	0.013245	0.1456954	0.01545254	0.0419426	0.01103753	0.333333	0.081678	0.377483	0.161148	0.05298	0.889625	0.116998
Mike Huckabee	0.155886	0.149523	0.1134677	0.037116	0.1314952	0.012725	0.1569459	0.03075292	0.2036055	0.03181336	0.305408	0.150583	0.144221	0.187699	0.235419	0.7614	0.26193
Scott Walker	0.162353	0.124706	0.09647059	0.009412	0.1741176	0.011765	0.3388235	0.02352941	0.05411765	0.02352941	0.287059	0.105882	0.185882	0.362353	0.077647	0.825882	0.192941
Lindsey Graham	0.132505	0.281574	0.04347826	0.008282	0.2815735	0.014493	0.1625259	0.00621118	0.05486542	0.02898551	0.414079	0.05176	0.296066	0.168737	0.083851	0.674948	0.339545
Bobby Jindal	0.24228	0.099762	0.0783848	0.026128	0.1235154	0.014252	0.192399	0.03800475	0.1852732	0.02137767	0.342043	0.104513	0.137767	0.230404	0.206651	0.821853	0.199525
Rick Santorum	0.148781	0.097561	0.06341463	0.019512	0.3268293	0.02439	0.1292683	0.02195122	0.1609756	0.02682927	0.246341	0.082927	0.35122	0.15122	0.187805	0.829268	0.190244
George Pataki	0.097493	0.21727	0.1114206	0.005571	0.2005571	0.022284	0.2172702	0.01114206	0.1225627	0.03064067	0.314763	0.116992	0.222841	0.228412	0.153203	0.749304	0.286908
Rick Perry	OUTLIER																
**Average**	**0.178094**	**0.128684**	**0.076209383**	**0.022141**	**0.23058491**	**0.012734**	**0.203576271**	**0.027029314**	**0.11145624**	**0.028167616**	**0.306778**	**0.098351**	**0.243319**	**0.230606**	**0.139624**	**0.799921**	**0.218757**
**Top 9 Average**	**0.194252**	**0.103898**	**0.070036848**	**0.025495**	**0.24876258**	**0.009796**	**0.206604375**	**0.030852358**	**0.09737342**	**0.028896343**	**0.29815**	**0.095532**	**0.258559**	**0.237457**	**0.12627**	**0.817029**	**0.198938**

Weighted Proportion of Moral Language: 2016 & 2020 General Election

Candidate	care.virtue	care.vice	fairness.virtue	fairness.vice	loyalty.virtue	loyalty.vice	authority.virtue	authority.vice	sanctity.virtue	sanctity.vice	Care	Fairness	Loyalty	Authority	Sanctity	Prop. Virtue	Prop Vice
Hillary Clinton 2016	0.253514	0.154054	0.1227027	0.064865	0.2064865	0.002162	0.132973	0.01837838	0.04486486	0.0227027	0.407568	0.187568	0.208649	0.151351	0.067568	0.760541	0.262162
Donald Trump 2016	0.161712	0.155767	0.07015458	0.135553	0.1700357	0.010702	0.1783591	0.03804994	0.04399524	0.06064209	0.317479	0.205707	0.180737	0.216409	0.104637	0.624257	0.400713
Joe Biden 2020	0.275029	0.101675	0.09583171	0.021815	0.2559408	0.002727	0.08648228	0.01636151	0.07713284	0.07791196	0.376704	0.117647	0.258668	0.102844	0.155045	0.790417	0.220491
Donald Trump 2020	0.186727	0.111133	0.08207343	0.064598	0.1590418	0.01237	0.1992931	0.05438838	0.05988612	0.09346161	0.29786	0.146672	0.171412	0.253681	0.153348	0.687021	0.335951
**Average**	**0.219245**	**0.130657**	**0.092690605**	**0.071708**	**0.1978762**	**0.00699**	**0.14927687**	**0.031794553**	**0.05646977**	**0.06367959**	**0.349903**	**0.164399**	**0.204866**	**0.181071**	**0.120149**	**0.715559**	**0.304829**
**Dem. Average**	**0.264271**	**0.127865**	**0.109267205**	**0.04334**	**0.23121365**	**0.002445**	**0.10972764**	**0.017369945**	**0.06099885**	**0.05030733**	**0.392136**	**0.152607**	**0.233658**	**0.127098**	**0.111306**	**0.775479**	**0.241327**
**Repub. Average**	**0.17422**	**0.13345**	**0.076114005**	**0.100076**	**0.16453875**	**0.011536**	**0.1888261**	**0.04621916**	**0.05194068**	**0.07705185**	**0.307669**	**0.17619**	**0.176074**	**0.235045**	**0.128993**	**0.655639**	**0.368332**

Table S8: Weighted/filtered proportions of moral language at the candidate level.

2.2 Customized moral foundations dictionary

Every word in the list below was used at least three times by a single candidate and achieved a *tf-idf* frequency score of <.0014.

Care Virtue

healthcare, child, safety, loves, benefits, health, heal, sharing, healing, shared, helped, shares, compassion, protecting, protection, mother, healthy, help, patient, helps, relief, feed, vulnerable, benefit, protected, compassionate childhood, helping, patients, charity, cares, condolences, generous, safe, mothers, loved, healthier, protects, safely, kindness, nurses, hospital, loving, caring, care, mommy, childcare, empathy, share, humane, helpful, nursing, protective, wounded, mercy, rescue, comfort, wounds, comforted, hospitality, nurse, charitable, lover, generosity, relieve, feeds, feeding, wound, generously, healed, rescuing, healers, hug, safeguard

Care Vice

violence, destroy, kill, assault, threats, fights, killing, cruelty, cruel, pain, destroyed, fighter, suffering, vulnerable, killed, rape, destroying, hurt, damage, attacked, threatens, die, hurting, destruction, victims, violent, attack, hunger, harm, fighting, threaten, murdered, hurts, persecution, punch, fighters, bully, bullying, murder, threatening, attacks, threat, harassment, injured, suffer, killer, wounded, bullies, rapists, suffers, victim, genocidal, destroys, killers, abuses, crying, threatened, abusers, abused, wounds, suffered, endanger, harmful, abusing, exploit, distressed, brutality, damaging, exploiting, torture, harms, kills, tribulation, injury, endangered, punches, agony, wound, endangering, bullied, assaulting, needy, carnage, harsh, harmed, assassination, genocide, exploitation

Fairness Virtue

equality, rights, equal, justice, integrity, reparations law, equity, fair, honesty, trust, laws, honest, trusted, justices, lawyer, fairness, lawyers, equitable, compensation parity, equalizers karma, compensate, trusting, repay, justify, retaliate,

Fairness Vice

dishonest, fraud, biased, racism, racist, discrimination, lying, inequality, lied, liar, sexist, unfair, scam, stealing, injustice, disproportionately, crooked, hypocrite, theft, cheating, bias, fraudulent, unjust, hypocrisy, liars, oppression, injustices, segregation, stole, hypocrites, freeloading, bigoted, betrayal, sexism, cheat, segregated, prejudice, robbing, exploit, racists, discriminate, exploiting, inequities, betray, betrayed, misleading, disparity, crooks, discriminating, deception, discriminated, cheated, defrauded, unequal, stolen, steal, scammed, cheats, biases, crook, exploitation, scams, imbalances, robbed

Loyalty Virtue

communities, community, homeland, patriot, coalition, troops, companies, wife, unite, joining, groups, followers, allies, patriots, countries, belonging, unity, fellow, pledge, company, nation, nations, group, united, ally, sacrifice, belong, organization tribal, pledged, solidarity, war, uniting, collective, belongs, corps, sacrifices, together, organizations, unites, allegiance, loyal, uniter, collectively, familiar, insider, follower, sacrificed, allied, pledges, player, insiders, coalitions, tribe, enlist, indivisible, tribes, loyalty, pledging, cohorts, fellowship, cult, troop

Loyalty Vice
enemy, enemies, outsider, disloyal, traitor, rebels, betray, betrayed, treason

Authority Virtue
bosses, commander, governor, chief, police, governors, dictate, regulations, ruling, submit, respected, order, traditional, willing, captains, protecting, protection, duty, pope, father, authority, ranked, control, govern, controlled, institutions, policing, fathers, respect, servant, presidents, punish, command, protects, manager, ceo, tradition, dominate, honored, governing, regulation, worship, bully, allegiance, elderly, bullying, arrested, proper, managers, honor, dominating, institution, rank, dictators, institutional, commandments, admiral, honoring, submitted, submissions, captain, dictator, guide, dictating, oligarchy, bullies, punitive, boss, leader, respects, dictated, arrest, ordering, ordered, noble, authorities, dominant, polite, submission, obey, submitting, dominates, honors, respecting, punished, punishment, submits, arrests, chiefs, guiding, punishing, dictates, acquiesce, ranking, dean, bullied, slaves, honorable, governed, servants, elders, dominated, principal, mentor, permission, matriarch, comply, respectfully

Authority Vice
illegal, illegals, chaos, refuse, refuses, anarchists, lawless, refused, orders, unlawful, refusing, dissidents, rioters, disrespect, riots, rebels, overthrow, traditions, rioting, anarchy, uprising, overthrown, disrespects, treason, disorder

Sanctity Virtue
god, marriage, religious, clean, lord, bless, food, prayer, sanctity, soul, church, dignity, christian, bible, christians, pray, praying, pastor, prayers, spiritual, blessed, pope, yogi, faith, religion, body, decency, sacred, blood, mary, holy, pure, catholics, married, worship, faithful, gods, religions, biblical, blessings, spirituality, blessing, jesus, churches, catholic, immunity, noble, nuns, synagogue, christ, enshrined, cleaning, atone, immune, divinity, foods, purity, cleaner, marry, wholesome, souls, righteous, angel, dignified, mosque, pristine, temple

Sanctity Vice
corrupt, corruption, pandemic, epidemic, hell, virus, drug, waste, drugs, sexual, addiction, spreading, degrade, dirty, disgusting, damn, wasted, corrupting, horrific, wasting, disease, rot, sin, horrifying, rotten, dirt, horror, corrupted, trash, degrading, viral, shit, diseases, contaminants, garbage, plague, alcoholism, sleazy, swear, plagued, stain, plagues, disgusted, fester, godless, damning, pandemics, addicted, incest, swore, contamination, infection, abhor, infected

2.4 Additional corpus statistics

The following section, illustrated in **Tables S9-S11,** contains additional summary statistics for each campaign assessed.

2020 DEMOCRATIC PRIMARY								
**Candidate**	**# Debates**	**Twitter Handle**	**Start Date**	**End Date**	**# Days**	**Source (Start Date)**	**Source (End Date)**	**# of Tweets**
Joe Biden	**11**	JoeBiden	5/18/19	4/8/20	320.00	http://www.xinhuanet.com/english/2019-05/19/c_138069905.htm	https://www.nytimes.com/2020/04/08/us/politics/bernie-sanders-drops-out.html	2948
Bernie Sanders	**11**	BernieSanders	3/2/19	4/8/20	396.00	https://abcnews.go.com/Politics/bernie-sanders-kicks-off-2020-campaign-rally-native/story?id=61411470	https://www.nytimes.com/2020/04/08/us/politics/bernie-sanders-drops-out.html	6804
Elizabeth Warren	**10**	ewarren	2/9/19	3/5/20	386.00	https://edition.cnn.com/2019/02/09/politics/elizabeth-warren-campaign-kickoff-massachusetts/index.html	https://www.nytimes.com/2020/03/05/us/politics/elizabeth-warren-drops-out.html	7733
Amy Klobuchar	**10**	amyklobuchar	2/10/19	3/2/20	382.00	https://www.mprnews.org/story/2019/02/10/amy-klobuchar-2020-presidential-race-announcement	https://www.nytimes.com/2020/03/02/us/politics/amy-klobuchar-drops-out.html	3073
Pete Buttigieg	**10**	PeteButtigieg	4/14/19	3/1/20	317.00	https://www.nytimes.com/2019/04/15/us/politics/pete-buttigieg-speech.html	https://edition.cnn.com/2020/03/01/politics/buttigieg-campaign/index.html	2994
Andrew Yang	**7**	AndrewYang	11/6/17	2/11/20	815.00	https://docquery.fec.gov/pdf/624/2017110690866	https://www.theverge.com/2020/2/11/21134021/andrew-	14413

						11624/201711069086611624.pdf	yang-drops-out-2020-presidential-race-democratic	
Tom Steyer	**6**	TomSteyer	7/9/19	2/29/20	230.00	https://www.cnbc.com/2019/07/09/tom-steyer-billionaire-pushing-to-impeach-trump-changes-mind-and-decides-to-run-for-president.html	https://www.npr.org/2020/02/29/801952931/tom-steyer-to-drop-out-of-2020-presidential-race?t=1621001603321	3599
Kamala Harris	**5**	KamalaHarris	1/21/19	3/12/19	51.00	https://www.politico.com/story/2019/01/21/kamala-harris-2020-campaign-1116076	https://www.cnbc.com/2019/12/03/kamala-harris-drops-out-of-2020-presidential-race.html	3586
Cory Booker	**5**	CoryBooker	2/1/19	1/13/20	342.00	https://www.youtube.com/watch?v=mx5m6DDFupg	https://www.cbsnews.com/news/cory-booker-ends-campaign-presidential-race-today-2020-01-13/	2577
Tulsi Gabbard	**4**	TulsiGabbard	3/2/19	3/19/20	377.00	https://www.huffingtonpost.co.uk/entry/tulsi-gabbard-campaign-launch_n_5c56a229e4b0871047542569?ri18n=true	https://eu.usatoday.com/story/news/politics/elections/2020/03/19/tulsi-gabbard-ends-presidential-campaign/2009989001/	1772
Julian Castro	**4**	JulianCastro	1/13/19	1/2/20	349.00	https://edition.cnn.com/2019/01/12/politics/julian-castro-presidential-announcement/index.html	https://www.nytimes.com/2020/01/02/us/politics/julian-castro-dropping-out.html	4025
Beto O'Rourke	**4**	BetoORourke	3/14/19	11/1/19	227.00	https://www.politico.com/story/2019/03/14/beto-orourke-2020-	https://www.nytimes.com/2019/11/01/us/politics/beto-orourke-drops-out.html	3602

						president-1207704		
Michael Bloomberg	**2**	MikeBloomberg	11/24/19	3/5/20	101.00	https://www.nytimes.com/2019/11/24/us/politics/michael-bloomberg-2020-presidency.html	https://www.bbc.co.uk/news/world-us-canada-51742481	948
Michael Bennet	**2**	MichaelBennet	5/2/19	2/12/20	280.00	https://www.theguardian.com/us-news/2019/may/02/michael-bennet-2020-presidential-campaign	https://edition.cnn.com/2020/02/11/politics/michael-bennet-ends-2020-campaign/index.html	3023
John Delaney	**2**	JohnDelaney	7/28/17	1/30/20	902.00	https://www.youtube.com/watch?v=DCCzeFu-7Wg	https://www.nytimes.com/2020/01/31/us/politics/john-delaney-drops-out.html	5738
Marianne Williamson	**2**	marwilliamson	1/28/19	1/10/20	342.00	https://abcnews.go.com/Politics/marianne-williamson-oprah-confidant-author-spiritual-teacher-presidential/story?id=60709204	https://www.huffpost.com/entry/marianne-williamson-drops-out-presidential-race_n_5d66fe2ce4b01fcc690e7c7c	3729
Tim Ryan	**2**	TimRyan	4/4/19	10/24/19	200.00	https://eu.cincinnati.com/story/news/politics/2019/04/04/ohio-rep-tim-ryan-announces-presidential-run/3363536002/	https://edition.cnn.com/2019/10/24/politics/tim-ryan-ends-campaign/index.html	2017
Bill de Blasio	**2**	BilldeBlasio	5/16/19	9/20/19	124.00	https://edition.cnn.com/2019/05/15/politics/bill-de-blasio-2020-presidential-announcement/index.html	https://www.politico.com/story/2019/09/20/bill-de-blasio-ends-2020-presidential-campaign-1506011	898

Kirsten Gillibrand	**2**	SenGillibrand	3/17/19	8/28/19	161.00	https://www.nytimes.com/2019/03/17/us/politics/gillibrand-2020-announce.html		1598
Jay Inslee	**2**	JayInslee	3/1/19	8/22/19	171.00	https://edition.cnn.com/2019/03/01/politics/inslee-2020-presidential-campaign/index.html	https://www.business-standard.com/article/pti-stories/washington-gov-jay-inslee-says-he-s-ending-presidential-bid-119082200175_1.html	3175
John Hickenlooper	**2**	Hickenlooper	3/4/19	8/15/19	161.00	https://edition.cnn.com/2019/03/04/politics/john-hickenlooper-presidential-campaign/index.html	https://abcnews.go.com/Politics/john-hickenlooper-end-2020-white-house-bid/story?id=64974362	1265
							**Total Tweets:**	**83,274**
							**Avg. Tweets:**	**3,621**

**2016 DEMOCRATIC PRIMARY**								
**Candidate**	**Debates Qualified**	**Twitter Handle**	**Start Date**	**End Date**	**# Days**	**Source (Start Date)**	**Source (End Date)**	**# of Tweets**
Hillary Clinton	**9**	HillaryClinton	4/12/15	6/6/16	414	http://www.xinhuanet.com/english/2019-05/19/c_138069905.htm	https://www.nytimes.com/2020/04/08/us/politics/bernie-sanders-drops-out.html	5881
Bernie Sanders	**9**	BernieSanders	4/30/15	6/6/16	396	https://abcnews.go.com/Politics/bernie-sanders-kicks-off-2020-campaign-rally-native/story?id=61411470	https://www.nytimes.com/2020/04/08/us/politics/bernie-sanders-drops-out.html	7354
Martin O'Malley	**4**	MartinOMalley	5/30/15	2/1/16	241	https://edition.cnn.com/2019/02/09/politics/elizabeth-warren-campaign-kickoff-massachusetts/index.html	https://www.nytimes.com/2020/03/05/us/politics/elizabeth-warren-drops-out.html	2285

**Total Tweets:**	**15,520**
**Avg. Tweets:**	**5,173**

**2016 REPUBLICAN PRIMARY**						
**Candidate**	**Debates Qualified**	**Twitter Handle**	**Start Date**	**End Date**	**# Days**	**# of Tweets**
Donald Trump	**12**	realDonaldTrump	6/16/15	5/4/16	318.00	5525
Ted Cruz	**12**	tedcruz	3/23/15	5/3/16	400.00	7342
John Kasich	**12**	JohnKasich	7/21/15	5/4/16	283.00	3671
Marco Rubio	**12**	marcorubio	4/13/15	3/15/16	332.00	3236
Ben Carson	**10**	RealBenCarson	5/3/15	3/4/16	301.00	1975
Jeb Bush	**9**	JebBush	6/15/15	2/20/16	245.00	2523
Chris Christie	**7**	GovChristie	6/30/15	2/10/16	220.00	356
Carly Fiorina	**7**	CarlyFiorina	5/4/15	2/10/16	276.00	1299
Mike Huckabee	**7**	GovMikeHuckabee	5/5/15	2/1/16	266.00	1869
Rick Santorum	**7**	RickSantorum	5/27/15	2/3/16	246.00	1090
Rand Paul	**6**	RandPaul	4/7/15	2/3/16	296.00	5516
Lindsey Graham	**4**	LindseyGrahamSC	6/1/15	12/21/15	200.00	2516
Bobby Jindal	**4**	BobbyJindal	6/24/15	11/17/15	143.00	1145
George Pataki	**4**	GovernorPataki	5/28/15	12/29/15	211.00	1055
Scott Walker	**2**	ScottWalker	7/13/15	9/21/15	68.00	1227
					**Total Tweets:**	40345
					**Average Tweets:**	2689

**2016 & 2020 GENERAL ELECTION NOMINEES**						
**Candidate**	**Debates Qualified**	**Twitter Handle**	**Start Date**	**End Date**	**# Days**	**# of Tweets**
2016 Clinton	**N/A**	HillaryClinton	6/6/16	11/8/16	152	4110
2016 Trump	**N/A**	realDonaldTrump	5/4/16	11/8/16	184	2233
2020 Biden	**N/A**	JoeBiden	4/8/20	11/3/20	205	2286
2020 Trump	**N/A**	realDonaldTrump	3/11/20	11/3/20	232	8662

**Total Tweets:**	**17291**
**Avg. Tweets:**	**4323**

Tables S9-S11: Summary statistics for each campaign assessed.

Section 3: Supplementary Figures

The following section contains supplementary figures which offer some utility for interpretating the main manuscript. **Figure S6** contains word clouds of the most common language associated with each foundation. **Figure S7-S9** are larger versions of the networks displayed in the main network, suitable for closer viewing. **Figure S10** and **Figure S11** offer granular, candidate level distributions of moral language use.

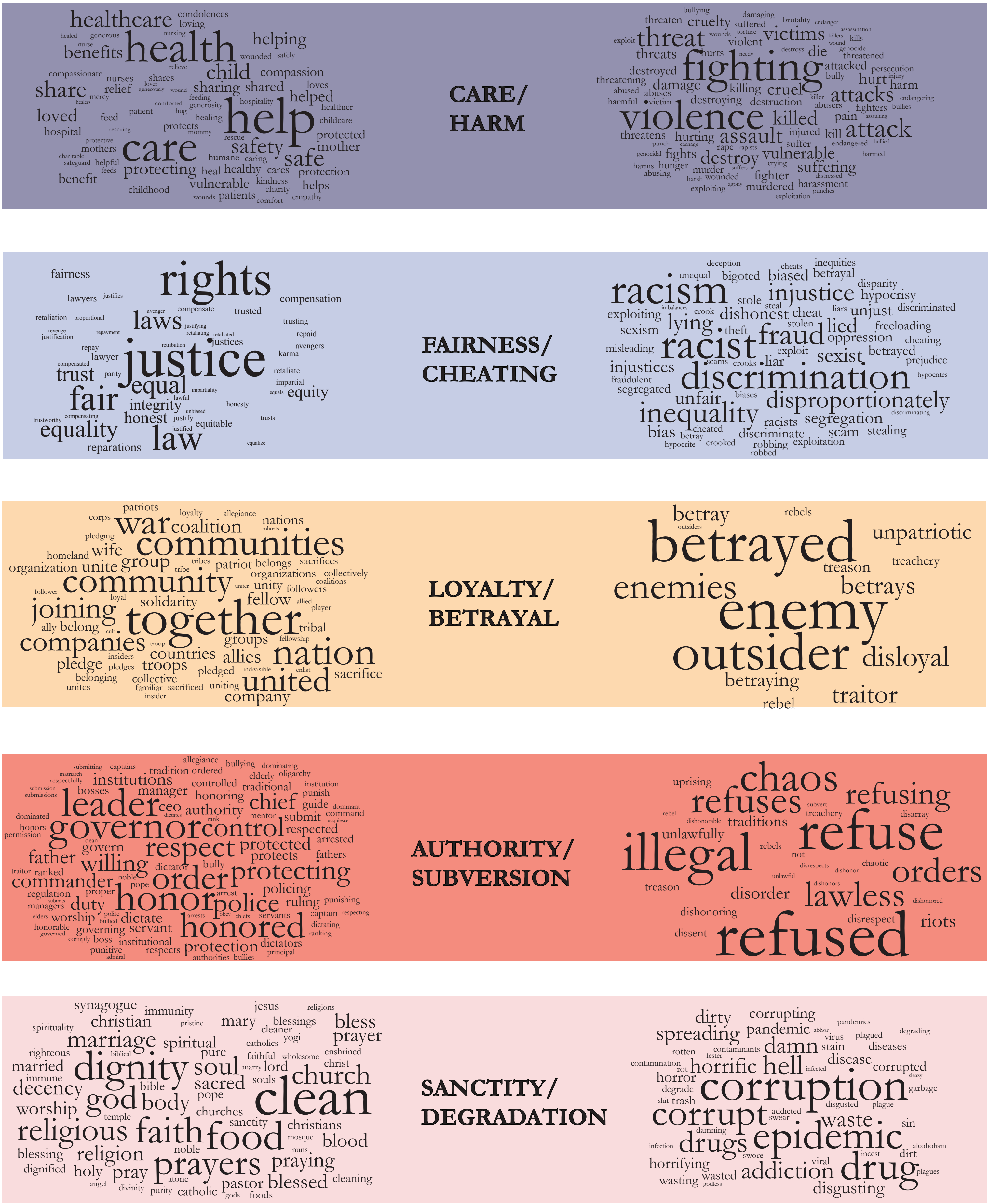

Figure S6 Word clouds of the extracted moral language most used by 39 U.S. presidential candidates on Twitter, 2016-2020. Word clouds generated from a corpus of 139,412 tweets; word size scaled with the square root of its occurrences.

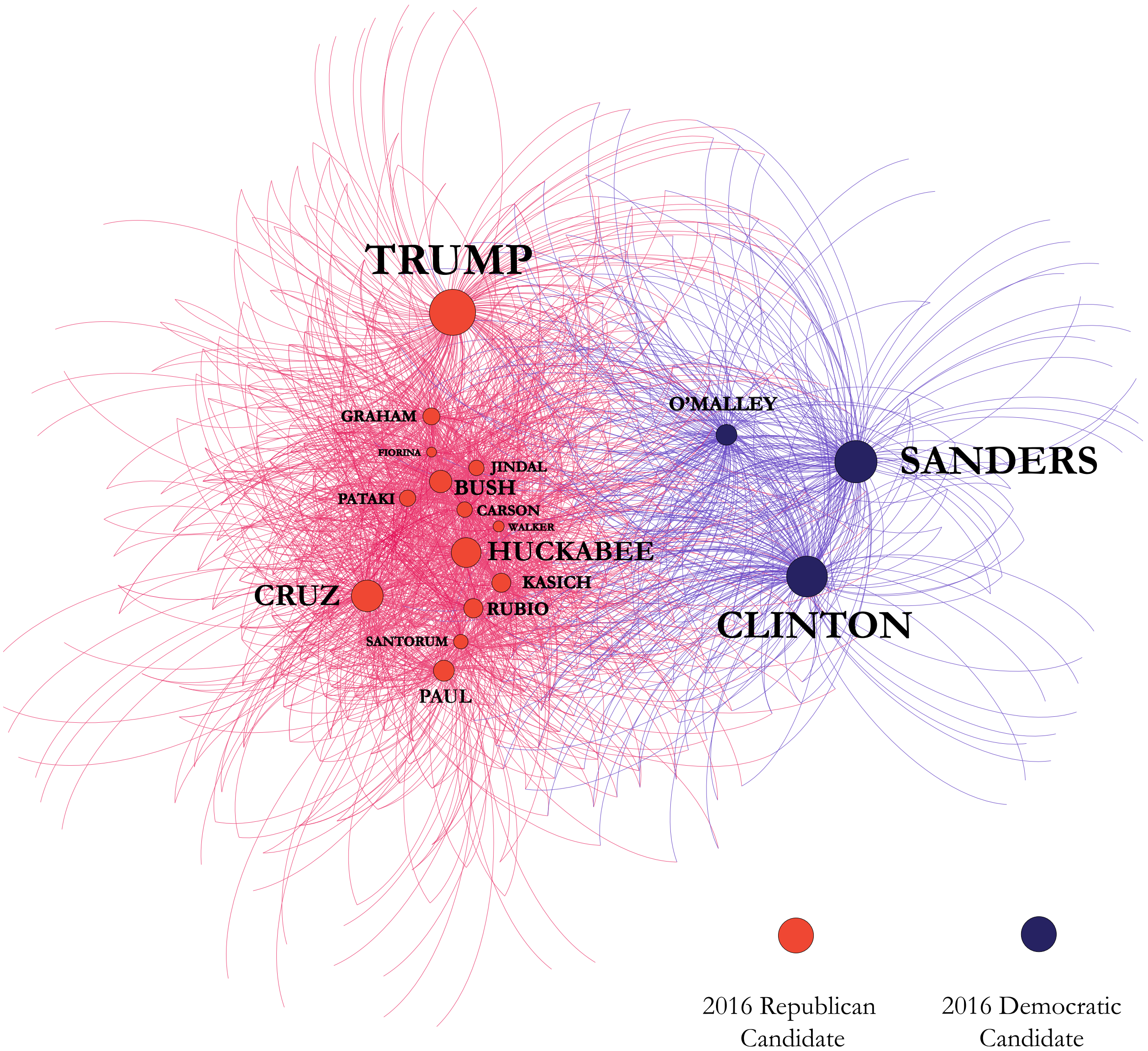

Figure S7 Bipartite text network displaying the moral-rhetorical community structure of the 2016 U.S. presidential primaries, based on a frequency analysis of 574 moral terms used by 17 Democratic and Republican candidates on Twitter. Nodes were colored using a Louvain community detection algorithm, which detected two communities perfectly reflecting partisan affiliation. Candidates are connected to each other through their use of the same moral language. Word nodes were removed to enhance readability, leaving spatialized candidate positions. Node and label sizes scale with betweenness centrality. Edges are colored by their candidate source node.

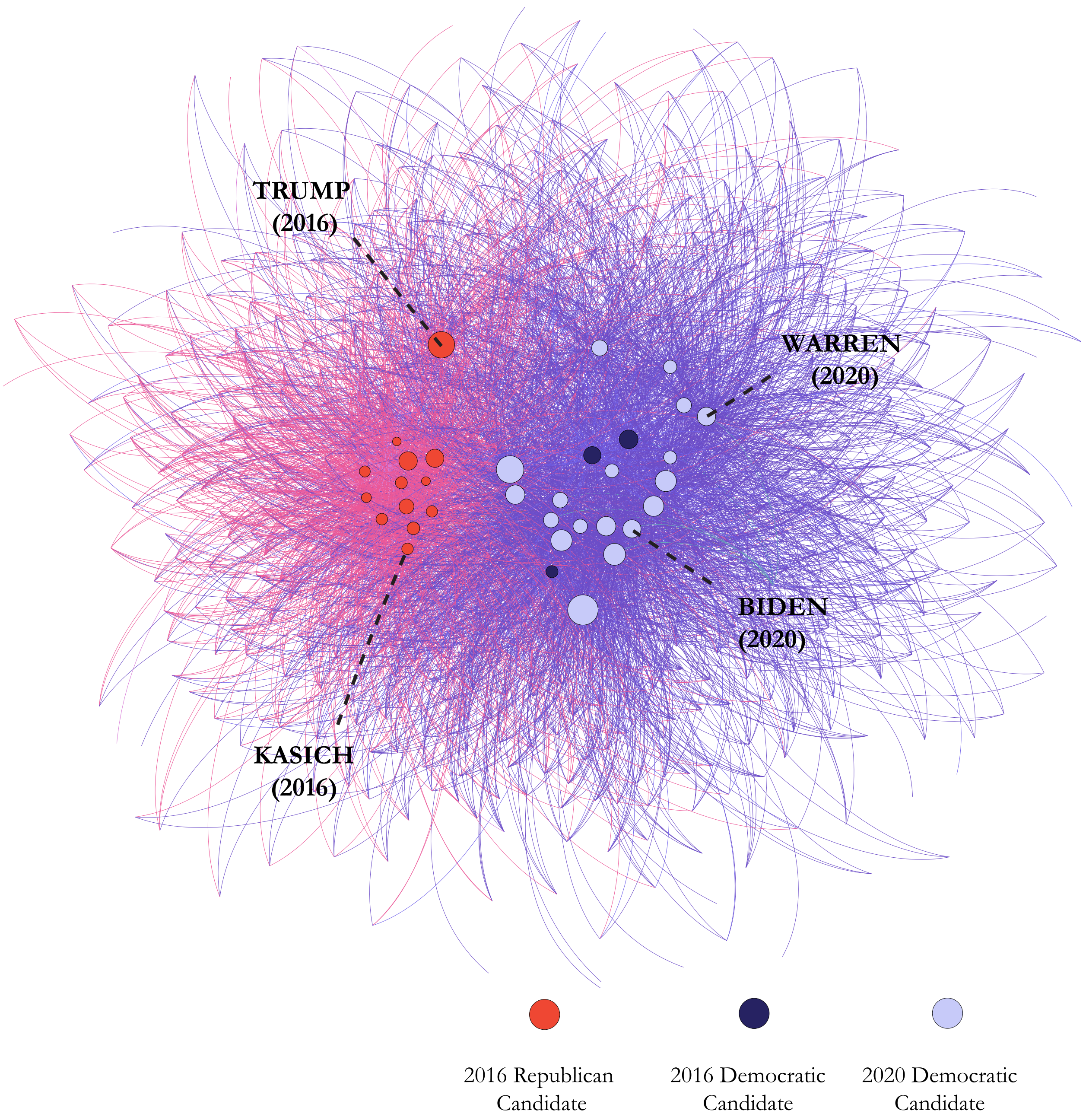

Figure S8 Bipartite text network displaying the moral-rhetorical community structure across both the 2016 and 2020 U.S. presidential primaries, based on a frequency analysis of 574 moral terms used by 39 Democratic and Republican candidates on Twitter. Candidates nodes are connected to each other through their use of the same moral language and colored by partisan affiliation; word nodes have been removed to enhance readability, leaving spatialized candidate positions. Node sizes are scaled by betweenness centrality. Edges connected to Democratic candidates are blue and edges connected to Republican candidates are pink. Some candidate labels were removed to enhance readability.

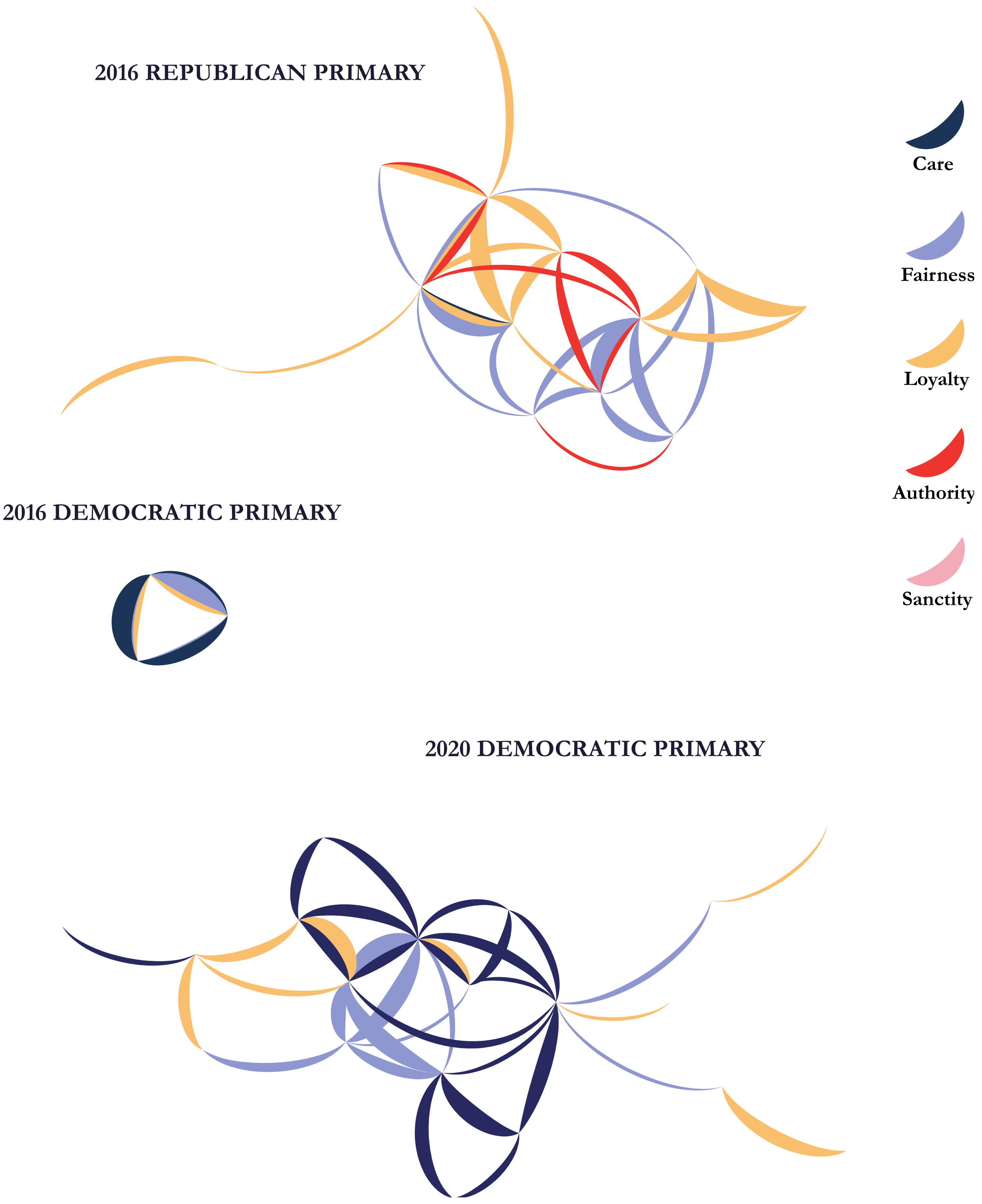

Figure S9 Network skeletons for each of three moral similarity networks. Nodes and candidate labels have been removed to highlight trends.

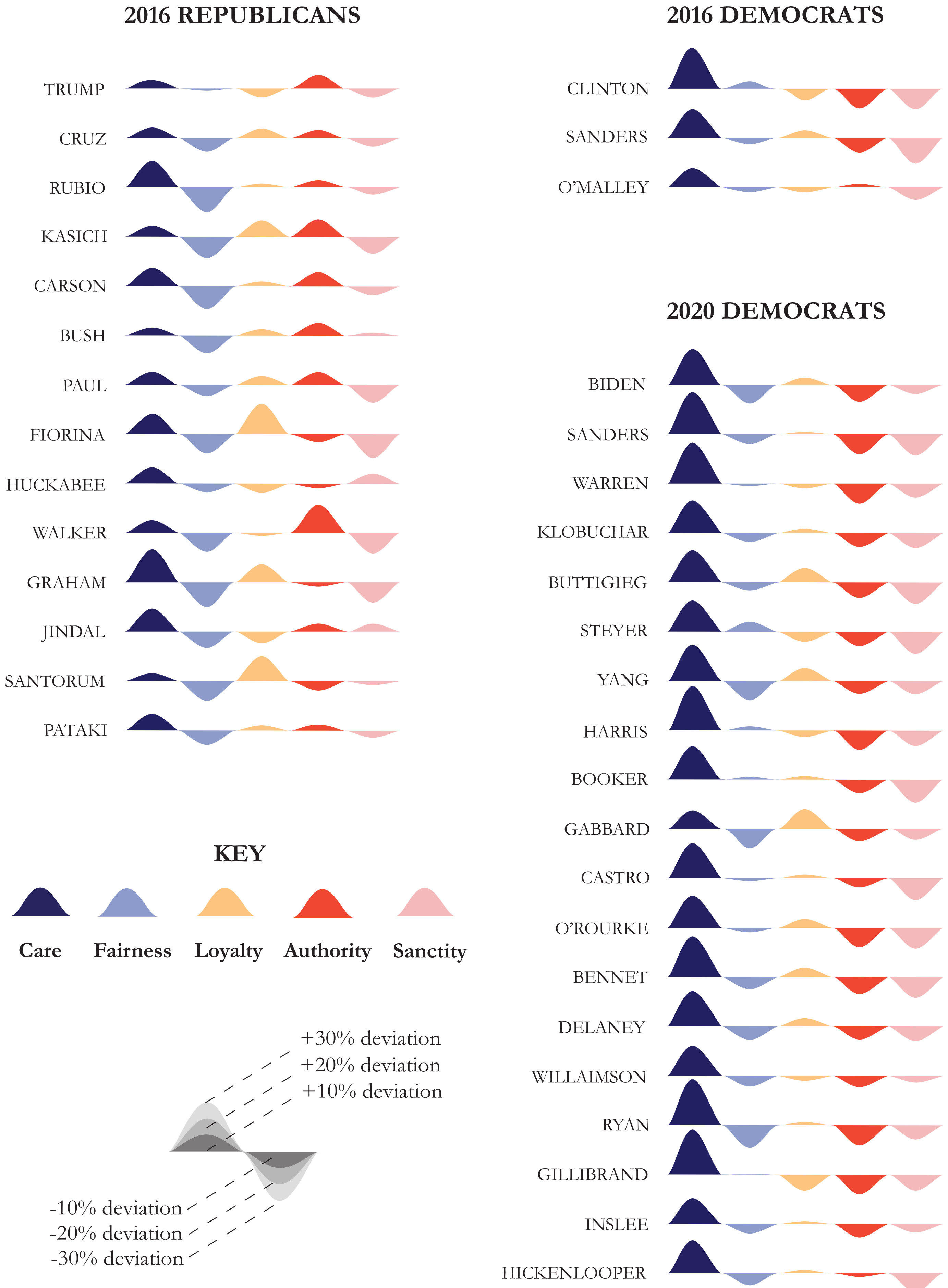

Figure S10 Deviation in proportion of moral foundation used by each candidate (deviations expressed in terms of deviation from 20%, the expected proportion if candidates used each moral foundation equally).

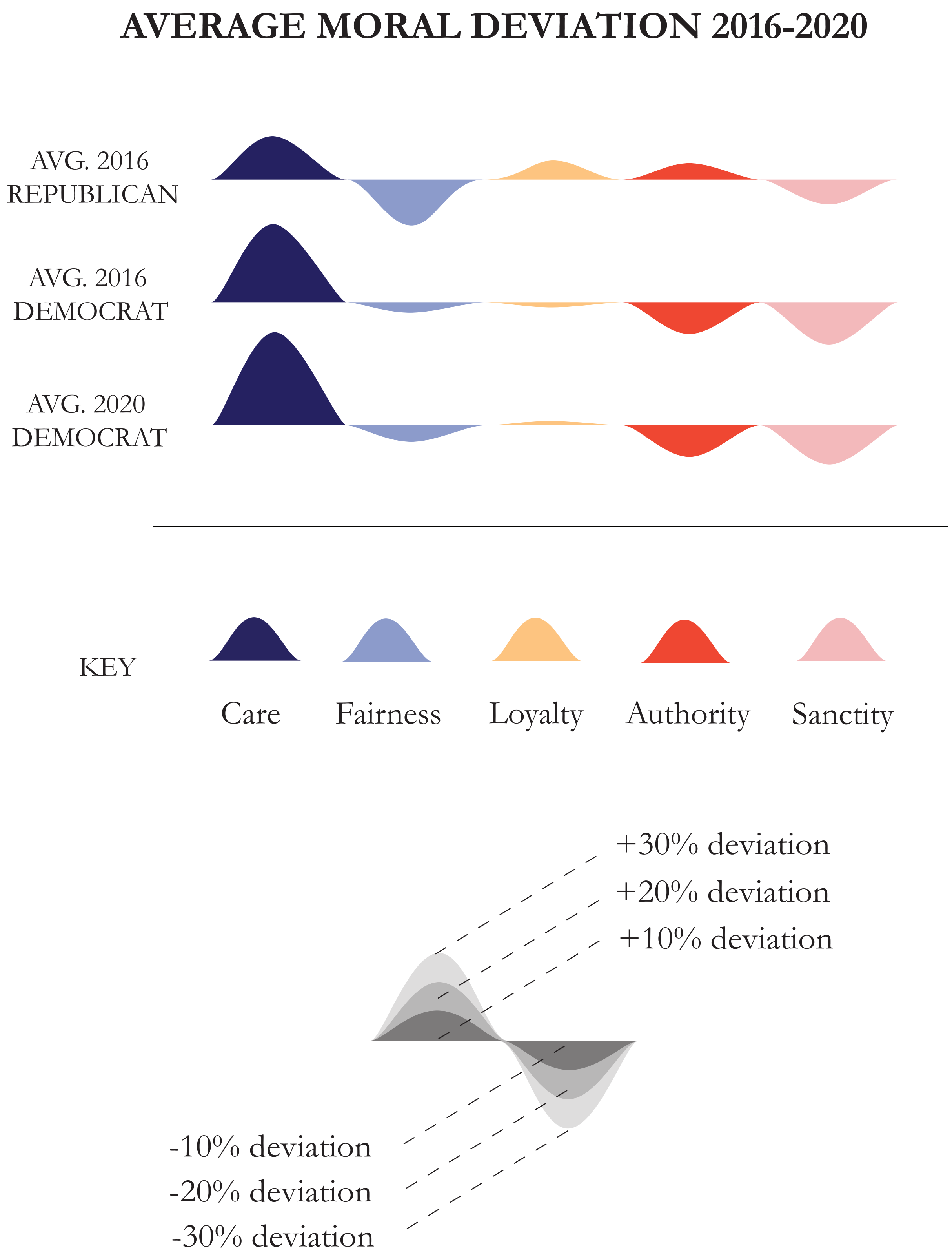

Figure S11 Deviation in proportion of moral foundation used by each party (deviations expressed in terms of deviation from 20%, the expected proportion if parties used each moral foundation equally).
